# Fine-Tuning of Optimal TCR Signaling in Tumor-Redirected CD8 T Cells by Distinct TCR Affinity-Mediated Mechanisms

**DOI:** 10.3389/fimmu.2017.01564

**Published:** 2017-11-15

**Authors:** Danilo Presotto, Efe Erdes, Minh Ngoc Duong, Mathilde Allard, Pierre-Olivier Regamey, Manfredo Quadroni, Marie-Agnès Doucey, Nathalie Rufer, Michael Hebeisen

**Affiliations:** ^1^Department of Oncology, Lausanne University Hospital Center, University of Lausanne, Lausanne, Switzerland; ^2^Protein Analysis Facility, Center for Integrative Genomics, University of Lausanne, Lausanne, Switzerland; ^3^Ludwig Institute for Cancer Research, University of Lausanne, Lausanne, Switzerland

**Keywords:** cancer immunotherapy, NY-ESO-1 antigen, T cell receptor engineering, T cell receptor-peptide-major histocompatibility complex affinity, hyporesponsiveness, CD3ζ, ERK1/2, SHP-1, SHP-2 phosphatases

## Abstract

Redirecting CD8 T cell immunity with self/tumor-specific affinity-matured T cell receptors (TCRs) is a promising approach for clinical adoptive T cell therapy, with the aim to improve treatment efficacy. Despite numerous functional-based studies, little is known about the characteristics of TCR signaling (i.e., intensity, duration, and amplification) and the regulatory mechanisms underlying optimal therapeutic T cell responses. Using a panel of human SUP-T1 and primary CD8 T cells engineered with incremental affinity TCRs against the cancer-testis antigen NY-ESO-1, we found that upon activation, T cells with optimal-affinity TCRs generated intense and sustained proximal (CD3ζ, LCK) signals associated with distal (ERK1/2) amplification-gain and increased function. In contrast, in T cells with very high affinity TCRs, signal initiation was rapid and strong yet only transient, resulting in poor MAPK activation and low proliferation potential even at high antigen stimulation dose. Under resting conditions, the levels of surface TCR/CD3ε, CD8β, and CD28 expression and of CD3ζ phosphorylation were significantly reduced in those hyporesponsive cells, suggesting the presence of TCR affinity-related activation thresholds. We also show that SHP phosphatases were involved along the TCR affinity gradient, but displayed spatially distinct regulatory roles. While PTPN6/SHP-1 phosphatase activity controlled TCR signaling initiation and subsequent amplification by counteracting CD3ζ and ERK1/2 phosphorylation, PTPN11/SHP-2 augmented MAPK activation without affecting proximal TCR signaling. Together, our findings indicate that optimal TCR signaling can be finely tuned by TCR affinity-dependent SHP-1 and SHP-2 activity, and this may readily be determined at the TCR/CD3 complex level. We propose that these TCR affinity-associated regulations represent potential protective mechanisms preventing high affinity TCR-mediated autoimmune diseases.

## Introduction

Adoptive transfer of tumor-reactive T cells has demonstrated that cytotoxic CD8 T cells are capable of mounting durable and protective immune responses against cancer. Owing to mechanisms of central tolerance, most of the self/tumor-specific T cells recognize their target with relatively low affinity/avidity compared to T cells recognizing non-self-antigens (1). Genetic engineering of cytotoxic CD8 T lymphocytes with affinity-optimized tumor-specific T cell receptors (TCRs) represents an interesting option for optimizing adoptive T cell transfer therapy. However, while therapeutic T cells should have maximal anti-tumor cytotoxicity and low side effects, such affinity-improved T cells may also induce on-target autoimmune reactivity against normal tissues expressing the same self-antigens (2). It is therefore important to improve our knowledge about the TCR affinity-mediated fine-tuning of T cell responses.

CD8 T cell responses are modulated by both the affinity of the TCR for its agonist pMHC complex and by the dose (density) of antigen present during T cell priming and activation. Many experimental evidences, including clinical trials, have highlighted that at physiologically low antigen dose, maximal T cell function occurs within a window of optimal TCR-pMHC affinity/half-lives, with short or long interactions adversely impacting on T cell function (3–10). Specifically, using a panel of engineered primary CD8 T cells expressing TCRs of incremental affinities for the NY-ESO-1 antigen, we found that T cells with supraphysiologically very high affinity TCRs lying above the optimal affinity window (*K*_D_ between 5 and 1 µM) were functionally impaired (3, 11, 12). This impairment could be gradually restored by augmenting the dose of antigen stimulation (3). Recent investigations have shown that pMHC dose and affinity for the TCR determine T cell responses by triggering common (e.g., for proliferation) but also distinct (e.g., IL-2 expression) gene expression programs downstream of the TCR signaling pathway (13). Modeling-based analyses on therapeutic TCR-transduced NY-ESO-1-specific T cells further enabled to unravel a minimal signaling architecture combining antigen affinity as well as dose effects (5). Altogether, these reports highlight that complex TCR affinity and antigen dose-associated regulatory mechanisms (positive and negative feedback loops) become integrated within the TCR signaling pathway following T cell activation.

Upon TCR triggering, TCR-mediated cell signaling occurs in a coordinated cascade of biochemical events. The Src-family kinase LCK is first recruited to the TCR complex to phosphorylate the immune-receptor tyrosine-based activating motifs (ITAMs) that are present on the various CD3 chains of the TCR complex, initiating signal transduction (14). Once fully phosphorylated, CD3/ITAMs recruit the Syk-family ZAP-70 kinase, which also becomes activated by LCK. Active ZAP-70 next phosphorylates the trans-membrane protein LAT and SLP-76, forming a signalosome scaffold enabling diversification of the signal into several branches, including the more distal MAPK signaling module (15). Controlled expression and activity of TCR/CD3 molecules, kinases and phosphatases represent a central strategy exploited by T cells to calibrate their activation threshold during development (16) and to produce a scaled T cell response, preventing immune overreactions and autoimmunity during inflammatory responses (17).

PTPN6/SHP-1 and PTPN11/SHP-2 are two important phosphatases that can be recruited by cell surface inhibitory receptors such as PD-1 or CTLA-4 (18). Mice with loss-of-function mutation in SHP-1 develop the motheaten phenotype associated with a severe inflammatory disorder (19), whereas specific deletion of SHP-1 in T cells enhances their proliferation potential (20). SHP-1 has been shown to globally counteract lymphocyte activation, notably by targeting proximal TCR signaling molecules such as LCK, ITAM/CD3ζ, LAT, and ZAP-70 (18). Unlike SHP-1, SHP-2 has been generally described as a positive regulator of T cell signaling (21), by sustaining the proliferative responses through indirect MAPK/Ras activation (22). However, an inhibitory effect for SHP-2 in controlling proximal TCR signaling has also been demonstrated (23). Notably, it recently became clear that SHP-2 preferentially dephosphorylate the CD28 T cell costimulatory receptor instead of the TCR to suppress T cell function, in response to PD-1 activation by PDL1 (24). As such, SHP-1 and SHP-2 are key players involved in signaling regulatory loops, adjusting the activation threshold during development (positive and negative selection) and calibrating peripheral T cell responses according to the nature and the affinity of the antigen (18). We previously reported that pharmacological inhibition of SHP-1 activity improved T cell function in all TCR-engineered T cells, and this correlated with the levels of SHP-1 phosphorylation along the TCR affinity gradient (25). Despite major efforts to understand how TCR-pMHC affinity modulates T cell specificity, function and on- or off-target toxicity (26), little is known about the TCR signaling characteristics of affinity-optimized T cells and the regulatory mechanisms (e.g., SHP-1 and SHP-2) involved in controlling T cell responsiveness according to affinity.

Here, we aimed at spatially and temporally dissect the impact of TCR-pMHC affinity on the signal intensity, amplification-gain and duration of the proximal (i.e., CD3ζ and LCK) and distal (i.e., ERK1/2) TCR signaling nodes. We further investigated the role of SHP-1 and SHP-2 phosphatases on those early TCR signaling events to evaluate their potential regulatory function. Collectively, optimal TCR affinity-mediated signaling depends on the fine-tuned intensity and duration of ITAM/CD3ζ and LCK phosphorylation levels after TCR triggering, associated with greatest pCD3ζ to pERK amplification-gain. We also found that TCR-ligand affinity modulates the levels of the TCR/CD3ε complex, CD8β and CD28 expression as well as of phosphorylated ITAM/CD3ζ readily under steady-state conditions. Moreover, optimal TCR signaling, leading to maximal function is calibrated in part by both SHP-1 and SHP-2 activity, which play distinct roles in the TCR signaling transduction cascade. These observations should help to define the best TCR signaling-mediated characteristics and promote the use of therapeutic affinity-engineered CD8 T cells.

## Materials and Methods

### Ethics Approval

Human peripheral blood cells were obtained from healthy donors of the Blood Transfusion Center of the University of Lausanne. All donors had previously completed the Swiss National Medical questionnaire to verify that they fulfilled the criteria for blood donation and provided written informed consent for the use of blood samples in medical research after anonymization.

### Cell Lines and Primary CD8 T Lymphocytes

T cell receptor α knock-out HLA-A68^pos^ (a member of the HLA-A2 supertype) (27) SUP-T1 cells (ATCC CRL-1942) and HLA-A2^pos^/TAP-deficient T2 cells (ATCC CRL-1992) were cultured at 37°C and 5% CO_2_ in RPMI 1640 supplemented with 10% FCS, 10 mM HEPES, penicillin (100 U/ml), and streptomycin (100 µg/ml). Human primary HLA-A2^pos^ or HLA-A2^neg^ CD8 T lymphocytes were obtained from peripheral blood mononuclear cells (PBMCs) from healthy donors following positive enrichment using anti-CD8-coated magnetic microbeads (Miltenyi Biotec), and cultured at 37°C and 5% CO_2_ in RPMI supplemented with 8% human serum and 150 U/ml recombinant human IL-2 (rhIL-2, gift from GlaxoSmithKline). CD8 T lymphocytes were expanded by periodic (every 14–21 days) restimulation with 1 µM PHA (Oxoid) and 30 Gy irradiated allogeneic PBMC as feeder cells.

### Lentiviral Production and Cell Transduction

Cloning strategies and lentiviral production were performed as described previously (3, 11). The full-length codon-optimized TCR AV23.1 and TCR BV13.1 chain sequences of a dominant NY-ESO-1_157–165_-specific T cell clone of patient LAU155 were cloned in the pRRL, third generation lentiviral vectors, as hPGK-AV23.1-IRES-BV13.1 or hPGK-AV23.1-T2A-BV13.1 constructs. The T2A construct was further modified by introducing a (T48C) and a (S57C) point mutation in the alpha and beta TCR constant region, respectively, to enhance preferential pairing (28). Structure-based amino acid substitutions were introduced into the wild-type (WT) TCR sequence using the QuickChange mutagenesis kit (Stratagene) and all mutations were confirmed by DNA sequencing. Concentrated supernatant of lentiviral transfected 293T/17 cells were used to infect SUP-T1 (15 min at 37°C) or primary CD8 T lymphocytes (overnight at 37°C). PE-labeled A2/NY-ESO-1_157–165_-specific multimers were used to sort transduced primary CD8 T cells in order to enrich for multimer-positive cells by flow cytometry (FACS Vantage SE machine; BD Biosciences). Integrated lentiviral copy number was relatively equivalent for each TCR variant and for each transduced cell type; i.e., 8–10 lentivirus copies/genome of SUP-T1 cells and 1–2 copies/genome of CD8 T cells (3).

### Flow Cytometry Analysis

For analysis of surface protein expression, 2.5 × 10^5^ TCR-transduced SUP-T1 or primary CD8 T cells were washed and stained with the following antibodies: panTCRαβ-PE, BV13.1-PE, TCR/CD3ε-FITC, and CD8β-FITC (Beckman Coulter). All experiments were performed under unstimulated, resting culture conditions. For total TCR/CD3ε protein expression analysis, unstimulated cells were fixed and permeabilized according to the phospho-flow assay protocol (see thereafter). Samples were acquired with a Gallios (Beckman Coulter) flow cytometer. FlowJo software (Tree star) and Prism software (GraphPad, USA) were used for data analyses.

### Phospho-Flow Assay

For phospho-flow staining experiments, 2.5 × 10^5^ TCR-transduced primary CD8 T cells or SUP-T1 cells were either left unstimulated or stimulated for the indicated period of time at 37°C with unlabeled A2/NY-ESO-1_157–165_ multimers (TC Metrix Sàrl, Switzerland) at final concentrations ranging from 0.001 to 100 µg/ml. For control experiments, TCR-transduced primary CD8 T cells or SUP-T1 cells were stimulated with 1 µg/ml PMA/250 ng/ml Ionomycin or with OKT3 anti-CD3ε antibody (10 µg/ml) for 5 min. The reaction was stopped immediately by fixing the cells with 4% paraformaldehyde (Polysciences) for 10 min at 37°C. Cells were permeabilized with 100% ice-cold methanol (Sigma Aldrich) for 20 min on ice and stained with the following antibodies: anti-phospho-ERK1/2 Alexa Fluor^®^ 647 (T202/Y204 of ERK1 and T185/Y187 of ERK2, Clone: E10, Cell Signaling Technology), antiphospho-CD3ζ (CD247) Alexa Fluor647 (Y142, Clone: K25-407.6, BD Phosflow), and unconjugated antitotal SHP-1 (ID:Y476, GeneTex) for 30 min at room temperature. For total SHP-1 staining, samples were further stained with FITC-conjugated goat antirabbit IgG (BD Pharmingen) for 20 min at 4°C. Samples were acquired with a Gallios (Beckman Coulter) or an ImageStream Mark II (Merck) flow cytometer. Representative cell images [geometric mean fluorescence intensity (gMFI) values corresponding to the population average] from the ImageStream analysis were blindly chosen by an independent collaborator. For the data analyses, FlowJo software (Tree star) and Prism software (GraphPad, USA) were used.

### Western Blot Analysis

For Western blot analyses, 1 × 10^6^ TCR-transduced SUP-T1 cells were either left unstimulated or stimulated for the indicated time points with unlabeled A2/NY-ESO-1_157–165_ multimers (TC Metrix Sàrl, Switzerland) at a final concentration of 1 µg/ml at 37°C. The reaction was stopped *via* ice-cold phosphate-buffered saline (PBS) and cells were lysed with RIPA buffer containing protease and phosphatase inhibitors (cOmplete and PhosSTOP, Roche). Protein quantification of the lysates was done using the Pierce BCA Protein Assay (Thermo Fisher Scientific, USA). 5–20 µg of denatured and reduced protein lysates were separated by SDS-PAGE followed by wet electrotransfer to nitrocellulose membranes. Membranes were blocked with 5% BSA or dry milk (in TBS-0.1% Tween 20) for 1 h at room temperature and subsequently immunoblotted overnight at 4°C. The following primary antibodies were used: antiphospho-CD3ζ (CD247) (pY142, clone K25-407.6; BD Pharmingen) and antiphospho-SHP-2 (Tyr580, clone D66F10, Cell Signaling Technology). Membranes were stained with HRP-conjugated species-specific secondary Abs for 1 h at room temperature. Anti-α-tubulin (B-5-1-2, Sigma Aldrich) was used as a loading control. The chemiluminescent signal was revealed by Western Bright ECL HRP substrate (Advansta) and images were acquired either with the Fusion FX (Vilber Lourmat) imaging system or by exposing the membrane to an X-ray film (Amersham Hyperfilm ECL, General Electric).

### Reverse-Phase Protein Assays (RPPA)

For all experiments, 1 × 10^6^ TCR-transduced SUP-T1 cells were resuspended in 100 µl complete prewarmed RPMI medium, and either left unstimulated (baseline) or stimulated with 100 µl prewarmed RPMI complete medium containing unlabeled A2/NY-ESO-1_157–165_ multimers at a final concentration of 1 µg/ml for 30 s or 1, 2, or 5 min. To stop the reaction, the cells were immediately put on ice, washed with 5 ml ice-cold PBS with freshly prepared protease and phosphatase inhibitors (cOmplete and PhosSTOP, Roche), and centrifuged in a cold bench top centrifuge for 5 min at 450 g. Supernatant was discarded and protein extracts were obtained by gently resuspending the cell pellets on ice for 40 min with 40 µl ZeptoMARK CLB-96 lysis buffer (3.5 M urea, 1 M thiourea, 0.4% CHAPS, 0.1% DTT, 5% DMSO, 10% glycerol, 0.4 mM spermidin, 0.2% pharmalyte, 1% octil-β-glucoside, and 1 mM sodium orthovanadate) containing freshly added protease and phosphatase inhibitors (cOmplete and PhosSTOP, Roche). Upon lysis, cell lysates were quickly frozen on dry ice and put at −80°C.

Reverse-phase protein assays were performed essentially as described (29). Cell lysates were quantified and serially diluted with CLB-96 lysis buffer before being spotted on ZeptoMARK chips, washed with assay buffer CAB1 (50 mM imidazole, 100 mM NaCl, 0.1% Tween 20, 5% BSA), and stained overnight at room temperature with a primary antibody against the protein of interest: total anti-LCK/Src (clone D88), anti-pLCK/pSrc (Y394/Y416), and anti-pSHP-2 (Y580 or Y542) from Cell Signaling, total anti-SHP-1 (C-19) and total anti-SHP-2 (C-18) from Santa Cruz, and anti-pSHP-1 (Y536, SP1571) from ECM Biosciences. Chips were washed with assay buffer CAB1 and incubated for 1 h at room temperature in the dark with a corresponding (antimouse or antirabbit) fluorescently labeled secondary antibody for signal generation. The fluorescent (Alexafluor 647-labeled) antibody was washed out with assay buffer CAB1, and chips were subsequently scanned with a ZeptoREADER microarray reader (Zeptosens, Witterswil, Switzerland).

### Cell Proliferation Assay

Native NY-ESO-1_157–165_ peptides were preincubated for 1 h at room temperature with the disulfide-reducing agent TCEP (2 mM, Pierce Biotechnology). 30-Gy irradiated HLA-A*0201-positive PBMCs were pulsed 1 h at 37°C with the indicated concentration of NY-ESO-1_157–165_ peptide, washed and incubated with CellTraceViolet-stained (Thermo Fisher), TCR-transduced primary CD8 T cells at an E:T ratio of 1:2 in RPMI 1640 medium supplemented with 8% human serum and 50 U/ml human rIL-2 (gift of GlaxoSmithKline). After 7 days, cells were acquired on a Gallios (Beckman Coulter, CA, USA) flow cytometer. Analysis of the data was done with the FlowJo software (Tree Star).

### Pharmacological Inhibition of SHP-1 and SHP-2 Phosphatase Activities

For pharmacological SHP-1 and SHP-2 inhibition, 10^5^ TCR-transduced SUP-T1 or primary CD8 T cells were treated with sodium stibogluconate (SSG) (100 µg/ml Sb content; Santa Cruz Biotechnology, Santa Cruz, CA) for 3 days, a concentration expected to inhibit both phosphatase activities (30). Cells were then stimulated, fixed, permeabilized, and stained with pERK1/2 or pCD3ζ (Y142) antibodies using the phospho-flow assay and analyzed on a flow cytometer.

### CRISPR/Cas9 Constructs Targeting *SHP-1* and *SHP-2* Genes

The 20 nucleotide-single guide RNA (sgRNA) sequences against SHP-1 and SHP-2 were designed using http://CRISPR.mit.edu/ and selected for high-quality scores (above 90 and 70, respectively) to minimize off-target effects. The guide sequences for the targets were as follows: SHP-1 (sgRNA no 3–4 TGAGTTCTGGATCCGAATAT) and (sgRNA no 5–6 TCACCCTGGTTCTTGCGACT); SHP-2 (sgRNA no 9–10 GTGCGCACTGGTGATGACAA). An extra 5′ G nucleotide was added to all guide sequences to improve U6 promoter efficiency. sgRNAs were generated by annealing DNA oligos and subsequently ligated into a BsmBI-digested lentiCRISPR plasmid (Addgen #49535). LentiCRISPR-EGFP-6 (a gift from Feng Zhang—Addgene plasmid #51765) was used as a mock control. Plasmids expressing SHP-1-, SHP-2-, or control (EGFP)-specific CRISPR/Cas9 constructs were transfected in HEK 293T/17 cells (ATCC CRL-11268) using a standard calcium phosphate protocol for the production of lentiviral particles. Lentiviral-containing supernatant was harvested twice (24 and 48 h post-transfection), filtrated, and concentrated 300× by ultracentrifugation. 0.5 × 10^6^ SUP-T1 cells stably expressing the different NY-ESO-1-specific TCR variants were then transduced with concentrated lentiviral particles for 15 min at 37°C. Two days after transduction, newly transformed puromycin-resistant SUP-T1 cells were selected in puromycin-containing RPMI 10% FCS medium (1 µg/ml) for 7 days. The penetrance of the knock-out phenotype was verified by Western blot or flow cytometry before further usage.

### Statistical Analysis

Data were analyzed using the Prism software (v.6, GraphPad) by matched one-way ANOVA followed by Dunnett’s multiple comparisons, by matched, two-way ANOVA followed by Tukey’s multiple comparisons or by two-tailed, paired *t* tests. The associated *p* values at α = 0.05 as well as numbers of experiments and sample sizes are indicated throughout the manuscript.

## Results

### Intensity of ERK1/2 Activation following Antigen-Dependent or -Independent TCR/CD3 Triggering Depends on TCR-Ligand Affinity

**Figure 1:**
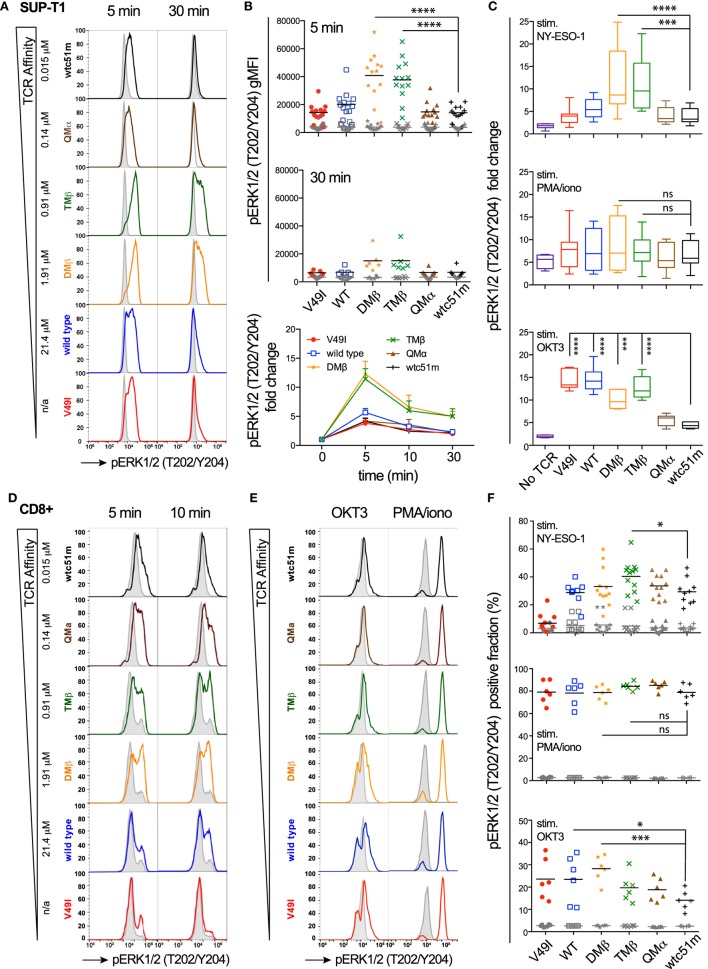
Temporal quantification of distal ERK1/2 phosphorylation levels in T cells engineered with T cell receptor (TCR) of incremental affinities. **(A)** Representative histograms of the phosphorylation levels of ERK1/2 by phospho-flow at baseline (gray) and the indicated time-points (color) of TCR-transduced SUP-T1 stimulated with NY-ESO-1-specific unlabeled multimers. *K*_D_ affinity values (μM) (3) for each TCR variant are indicated. **(B)** Quantification of pERK1/2 levels [geometric mean fluorescence intensity (gMFI)] for the TCR-transduced SUP-T1 variants (*n* > 6 independent experiments) at baseline, 5, 10, and 30 min poststimulation with NY-ESO-1-specific unlabeled multimers. **(C)** Relative intensity of ERK1/2 phosphorylation levels for the indicated TCR affinity variants (*n* = 6 independent experiments) after 5 min stimulation with NY-ESO-1-specific unlabeled multimers (top panel), PMA/ionomycin (middle), or OKT3 (anti-CD3ε) antibody (bottom). Data are depicted as box (25th and 75th percentile) and whisker (min to max) with the middle line representing the median. **(D)** Representative histograms of the phosphorylation levels of ERK1/2 by phospho-flow at baseline (gray) and at the indicated time-points (color) of TCR-transduced primary CD8 T cells stimulated with NY-ESO-1-specific unlabeled multimers. **(E)** Representative histograms of the phosphorylation levels of ERK1/2 by phospho-flow at baseline (gray) and at 5 min (color) of TCR-transduced primary CD8 T cells stimulated with OKT3 (anti-CD3ε) antibody or PMA/Ionomycin. **(F)** Positive fraction of ERK1/2 phosphorylation in primary CD8 T cells expressing the indicated TCR affinity variants (*n* ≥ 6 independent experiments) at baseline (gray) and after 5 min stimulation with NY-ESO-1-specific unlabeled multimers (top panel), PMA/ionomycin (middle) or OKT3 (anti-CD3ε) antibody (bottom). Statistical analyses were performed with matched, one-way ANOVA tests followed by Dunnett’s multiple comparisons. Significance of the adjusted *p* value at α = 0.05 is given by the following symbols: ns (*p* > 0.05) and *p ≤ 0.05, **p ≤ 0.01, ***p ≤ 0.001, ****p ≤ 0.0001. **(A–F)** ERK1/2 phosphorylation levels obtained by phospho-flow for each TCR variant are depicted by distinct symbols and color codes.

Using a panel of TCR-engineered A2/NY-ESO-1-specific SUP-T1 and primary CD8 T cells (3, 12), we first assessed the impact of TCR-pMHC affinity on the distal pERK1/2 signaling node in terms of signal intensity and duration by quantitative flow cytometry-based intracellular staining (i.e., phospho-flow) and RPPA. Baseline levels of pERK1/2 were low and similar for all TCR affinity variants (Figures 1A,B,D–F; Figure S1A in Supplementary Material). Upon antigen-specific TCR triggering with pMHC multimers, ERK1/2 phosphorylation displayed an on-off activation pattern at the population level, from pERK1/2^low^ to pERK1/2^high^ (Figures 1A,D,E), consistent with the previously reported switch-like digital response of ERK1/2 activation (31). We found strongest pERK1/2 levels and superior signal persistence in SUP-T1 and CD8 T cells expressing TCRs within the optimal affinity window (DMβ and TMβ), with a time-to-peak between 2 and 5 min (Figures 1B,D; Figure S1A in Supplementary Material). In contrast, T cells expressing very high affinity TCRs (QMα and wtc51m) exhibited both reduced ERK1/2 phosphorylation intensity and duration, resembling the ERK1/2 signaling pattern observed in T cells with lower affinity TCRs (V49I and wild-type). Yet, comparable levels of total ERK1/2 expression were observed for all TCR variants (Figure S1B in Supplementary Material).

We next asked whether this decline in ERK1/2 signaling is an intrinsic feature of T cells with very high affinity TCRs (hereafter called “very high affinity T cells”) or whether it highlights the presence of TCR affinity-dependent signal inhibitory mechanisms. To do so, SUP-T1 and primary CD8 T cells were stimulated in parallel experiments with either NY-ESO-1 multimers (antigen-specific/TCR-dependent) or OKT3 antibody (antigen-unspecific/TCR-dependent) or PMA/Ionomycin (antigen-unspecific/TCR-independent) (Figures 1C,E,F). Whereas antigen-specific TCR triggering with multimers recapitulated the typical bell-shape pattern, PMA/Ionomycin generated comparable ERK1/2 phosphorylation levels (i.e., fold change and fraction of pERK-responding cells) for each TCR variant, irrespective of TCR affinity. Interestingly, TCR crosslinking with the strong anti-CD3ε/OKT3 antibody produced an ERK1/2 phosphorylation pattern, which inversely correlated with TCR affinity. Very high affinity T cells showed again reduced ERK1/2 phosphorylation levels, while lower affinity T cells responded more vigorously to OKT3 (Figures 1C,E,F).

Extending on our previous report (25), these results show that the MAPK signaling node remains functional in all engineered SUP-T1 and primary CD8 T cell variants, with maximal ERK1/2 activation intensity and duration observed in T cells of optimal TCR affinities. However, ERK1/2 phosphorylation was reduced in the very high affinity T cells when induced by antigen-dependent or non-dependent TCR/CD3 triggering, suggesting the presence in those cells of negative regulators of the TCR signaling pathway. Conversely, the diminished ERK1/2 activation pattern observed in the low affinity V49I expressing T cells following antigen-specific but not antigen-independent TCR triggering is likely related to the poor intrinsic binding affinity of this particular TCR variant (3, 11).

### Optimal TCR Affinity-Mediated Signaling Depends on the Fine-Tuned Intensity and Duration of Phosphorylated ITAM/CD3ζ Levels

**Figure 2:**
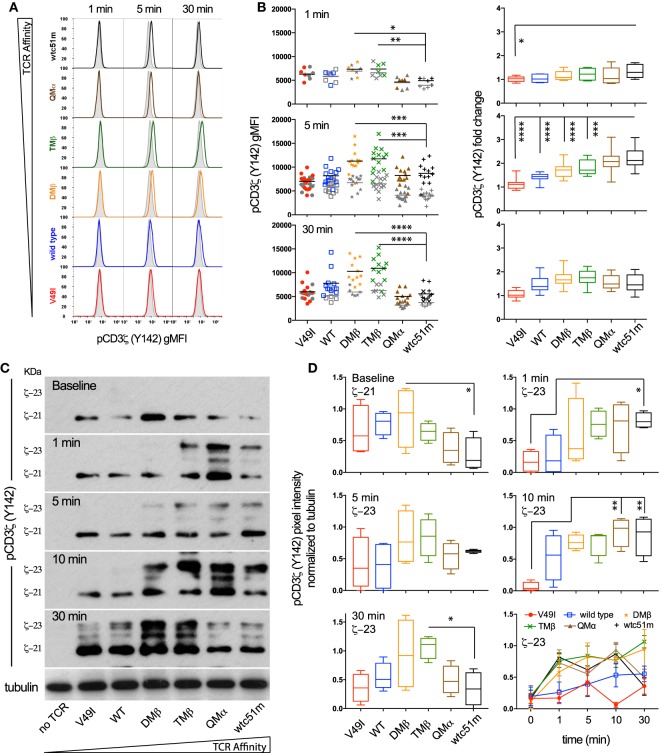
Temporal quantification of proximal CD3ζ phosphorylation levels in T cells engineered with T cell receptor (TCR) of incremental affinities. **(A)** Representative histograms of the phosphorylation levels of CD3**ζ** (Y142) at baseline (gray) and the indicated time-points (color) of TCR-transduced SUP-T1 variants stimulated with NY-ESO-1-specific unlabeled multimers. **(B)** Quantification of the absolute intensity [left panels, geometric mean fluorescence intensity (gMFI)] and relative intensity (right panels, fold change) of pCD3**ζ** (Y142) by phospho-flow for the indicated TCR-transduced SUP-T1 variants at 1, 5, and 30 min poststimulation with NY-ESO-1-specific unlabeled multimers (*n* > 10 independent experiments). **(C)** Levels of steady-state 21 kDa and fully activated 23 kDa immune-receptor tyrosine-based activating motif (ITAM)/pCD3**ζ** complexes of TCR-transduced SUP-T1 variants at baseline and at the indicated time-points after stimulation with NY-ESO-1-specific unlabeled multimers. Alpha-tubulin expression levels were used as loading controls between samples. Data are representative of four independent Western blot experiments. **(D)** Quantification of the relative pixel intensities of the baseline 21 kDa and fully activated 23 kDa ITAM/pCD3**ζ** bands detected by Western blot and normalized to α-tubulin in the TCR-transduced SUP-T1 variants at the indicated time-points after stimulation with NY-ESO-1-specific unlabeled multimers (*n* = 4 separate experiments). **(B,D)** Data are depicted as box (25th and 75th percentile) and whisker (min to max) with the middle line representing the median. Statistical analyses were performed with matched, one-way ANOVA tests followed by Dunnett’s multiple comparisons. Significance of the adjusted *p* value at α = 0.05 is given by the following symbols: ns *p* > 0.05 and *p ≤ 0.05, **p ≤ 0.01, ***p ≤ 0.001, ****p ≤ 0.0001. **(A,B,D)** Each TCR variant is depicted by a distinct symbol and color code.

To investigate whether the high TCR affinity-associated impairment of distal ERK1/2 phosphorylation is readily detectable at the proximal level of the TCR signaling cascade, we quantitatively assessed the intensity and duration of CD3ζ phosphorylation in SUP-T1 cell variants following antigen-specific stimulation (Figure 2). Upon TCR triggering, the relative amplitude (i.e., fold change between baseline and activated state) of pCD3ζ (Y142) phosphorylation gradually increased along the TCR affinity gradient, and was predominant in the supraphysiological T cells (QMα, wtc51m) at early time-points (Figures 2A,B). Accordingly, the very high affinity T cells were the first to generate fully active pCD3ζ 23 kDa complexes (Figures 2C,D), as depicted by the gel shift from 21 to 23 kDa (14). This trend progressively reversed over time in favor of the optimal affinity TCR variants (DMβ and TMβ), which displayed the highest intensity and longest duration of fully phosphorylated ITAM/CD3ζ 23 kDa (Figures 2C,D).

Using RPPA, we next quantified the phosphorylation levels of the activatory pLCK(Y394) form and found no changes for the baseline levels of pLCK or total LCK (Figures S2A,C in Supplementary Material) between the different TCR variants. Upon activation however, the levels of pLCK(Y394) rose rapidly (<30 s) in a TCR affinity-dependent manner from low to very high affinity TCR variants, thus preceding ITAM/CD3ζ phosphorylation with similar activation and kinetic patterns. No major changes were observed for the inhibitory pLCK(Y505) form at baseline or upon stimulation (Figure S2B in Supplementary Material). These observations indicate that despite its relatively high initial intensity, the proximal TCR signaling potential of very high affinity T cells remains transient. Thus, optimal TCR affinity-mediated signaling depends on the fine-tuned intensity and duration of ITAM/CD3ζ and LCK phosphorylation after TCR triggering.

### TCR-Ligand Affinity Modulates Expression and Phosphorylation Levels of the TCR/CD3 Complex Readily Under Steady-State Conditions

**Figure 3:**
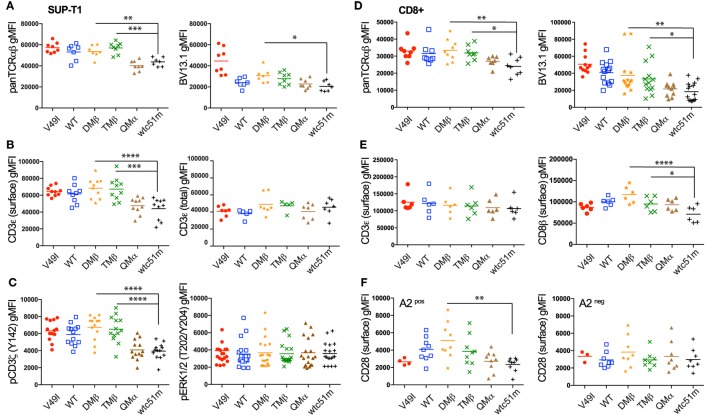
Baseline expression levels of the T cell receptor (TCR)/CD3 complex in SUP-T1 and primary CD8 T cells engineered with TCR of incremental affinities. **(A–C)** Quantification of the expression **(A–B)** and phosphorylation **(C)** levels [geometric mean fluorescence intensity (gMFI)] of unstimulated, baseline **(A)** pan TCRαβ and TRBV13.1, **(B)** extracellular CD3ε and total intra- and extracellular CD3ε, and (**C**) CD3**ζ** (Y142) and ERK1/2(T202/Y204) for the indicated TCR-transduced SUP-T1 variants. Data are representative of 5–20 independent experiments. **(D–E)** Quantification of the expression levels (gMFI) of unstimulated, baseline **(D)** pan TCRαβ and TRBV13.1 and **(E)** extracellular CD3ε and CD8β for the indicated TCR-transduced primary CD8 T cell variants. Data are representative of 6–15 independent experiments. **(F)** Quantification of the CD28 expression levels for the indicated TCR-transduced primary CD8 T cells isolated from an HLA-A2^pos^ (left panel) or an HLA-A2^neg^ (right panel) healthy donor under resting, unstimulated culture conditions. Data are representative of three to nine independent experiments **(A–F)** Statistical analyses were performed with matched, one-way ANOVA tests followed by Dunnett’s multiple comparisons. Significance of the adjusted *p* value at α = 0.05 is given by the following symbols: **p ≤ 0.01, ***p ≤ 0.001, ****p ≤ 0.0001. Each TCR variant is depicted by a distinct symbol and color code.

We previously observed that baseline surface expression of several activatory/costimulatory receptors (e.g., CD28, HVEM, and CD70) was significantly reduced in CD8 T cells of high TCR affinity (25). Here, we investigated whether the surface expression pattern of TCR/CD3 complex as well as the phosphorylation levels of CD3ζ and ERK1/2 could similarly be decreased in TCR-transduced SUP-T1 and newly engineered primary CD8 T cells under steady-state conditions, in the absence of antigenic stimulation. We observed that high affinity SUP-T1 cells (QMα and wtc51m) displayed substantially lower levels of surface TCRαβ and CD3ε as well as of phosphorylated ITAM/CD3ζ (Y142), while maintaining comparable expression levels of total (i.e., intracellular and extracellular) CD3ε complexes and pERK1/2 (Figures 3A–C). These data were confirmed by Western blot analyses (Figures 2C,D), showing in those cells reduced levels of pCD3ζ 21 kDa, a partially phosphorylated form of CD3ζ mostly found under resting culture conditions (32). Similar differences were found in the primary CD8 T cells with reduced levels of overall TCRαβ, TCR β-chain (BV13.1) and CD8β in very high affinity T cells (Figures 3D,E). A slight decline in surface levels of CD3ε could also be observed in these cells (Figure 3E), albeit baseline CD3ζ and ERK1/2 phosphorylation levels remained stable throughout the whole affinity panel (Figure S2D in Supplementary Material). The phenotypical variations observed between the SUP-T1 and the primary CD8 T cells might reflect the presence of the endogenous TCR/CD3 complexes expressed in the latter cells, but absent in the SUP-T1 cell line. Consistent with our previous report (25), lower expression of CD28 was found in supraphysiological CD8 T cells. In contrast, no marked decline in CD28 and TCR β-chain (BV13.1) expression was seen in TCR-engineered CD8 T cells lacking HLA-A2 expression (Figure 3F, preliminary unpublished data). Altogether, these results show that TCR-ligand affinity readily impacts on the expression levels of surface TCR/CD3ε, CD8β, and CD28 as well as of activated ITAM/CD3ζ at baseline, under resting conditions.

### Both TCR-Ligand Affinity and Antigen Dose Modulate the Strength and Amplification of the TCR-Mediated Signaling Pathway

**Figure 4:**
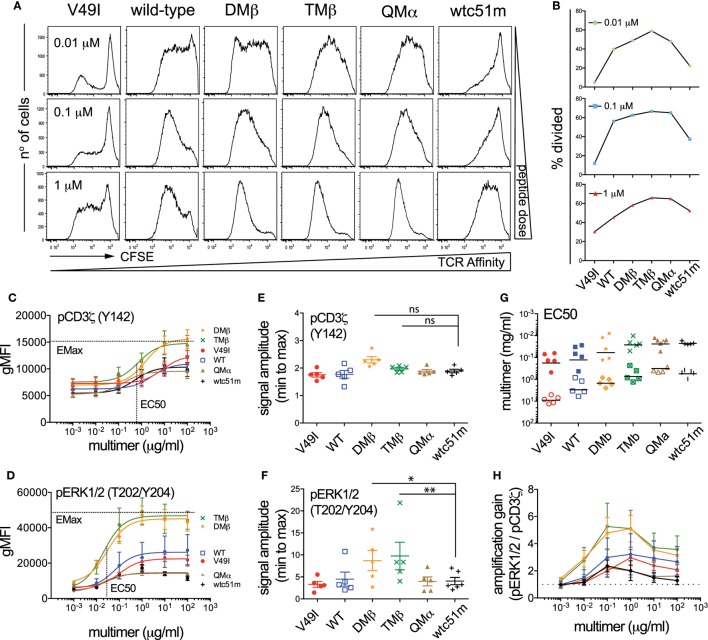
Effect of antigen dose on proliferation and T cell receptor (TCR)-mediated signal intensity and amplification of pCD3ζ and pERK1/2. **(A)** Proliferation analysis performed at day 7 for the indicated variant of CFSE-labeled, TCR-transduced primary CD8 T cells after stimulation with titrating doses of NY-ESO-1 antigen-pulsed irradiated PBMCs. **(B)** Quantification of the percentage of divided cells (frequency of dividing cells) from the proliferation histograms shown in **(A)**. Data obtained from the different antigen doses (0.01, 0.1, and 1 µM) are depicted as individual graphs. **(C,D)** Average phosphorylation levels of **(C)** CD3**ζ** (Y142) (*n* = 5 independent experiments) and **(D)** ERK1/2 (*n* = 5) by phospho-flow following stimulation at 5 min of the indicated TCR-transduced SUP-T1 variants with titrating doses of NY-ESO-1-specific unlabeled multimers. **(E,F)** Quantification of the signal amplitude (from minimal to maximal Emax level) of **(E)** pCD3**ζ** (Y142) and **(F)** pERK1/2 for the indicated TCR-transduced SUP-T1 variants. Statistical analyses were performed with matched, one-way ANOVA tests followed by Dunnett’s multiple comparisons. Significance of the adjusted *p* value at α = 0.05 is given by the following symbols: ns *p* > 0.05 and *p ≤ 0.05, **p ≤ 0.01. **(G)** Quantification of the EC50 antigen dose producing half of the maximal signal amplitude for pCD3**ζ** (Y142) (open symbols) and pERK1/2 (plain symbols) for the indicated TCR-transduced SUP-T1 variants. **(H)** Quantification of the signal amplification gain (relative fold increase) from pCD3**ζ** (Y142) to pERK1/2 following 5 min stimulation of the indicated TCR-transduced SUP-T1 variants with titrating doses of NY-ESO-1-specific unlabeled multimers. **(C–H)** Each TCR variant is depicted by a distinct symbol and color code.

T cell receptor stimulation with high doses of antigen can modulate T cell functional output in a TCR affinity-dependent manner (3, 5, 10, 33). In line with these previous reports, we found that enhanced density of NY-ESO-1 antigen increased the proportion of primary CD8 T cells capable to degranulate CD107a and produce cytokines, with the low (V49I) and very high (wtc51m) affinity T cells still requiring higher antigen doses than the other variants to reach maximum levels (Figure S3 in Supplementary Material). In contrast to the relative recovery in CD107a degranulation and cytokine secretion observed in the very high affinity CD8 T cells, both proliferation and expansion capacity of those cells remained reduced even at high, saturating antigen doses (Figures 4A,B). Thus, increase in antigen dose was unable to fully restore the proliferative capacity of the very high affinity primary CD8 T cells when compared to the optimal variants (DMβ and TMβ).

To gain further insights into the mechanisms underlying the impaired proliferative capacity associated with low and very high TCR affinity variants, we explored the impact of increased antigen doses on proximal (ITAM/pCD3ζ) and distal (pERK1/2) signaling efficiency (Figures 4C–H). We found that the phosphorylation pattern of CD3ζ steadily increased in each engineered SUP-T1 variant along the antigen dose, with absolute pCD3ζ (Y142) levels being higher in optimal affinity TCR variants (DMβ and TMβ) at all multimer concentrations (Figure 4C). Yet, the maximal amplitude of pCD3ζ signal (i.e., fold change from baseline to maximum activated levels) was comparable along the different TCR variants (Figure 4E). At the MAPK node, increasing the antigen dose also allowed discriminating different pERK1/2 phosphorylation levels (Figure 4D), but this time optimal affinity T cells presented significantly higher signal amplitude when compared to low (V49I, wild-type) and very high affinity (QMα, wtc51m) T cells (Figure 4F). As expected, the sensitivity of TCR triggering (i.e., EC_50_) similarly increased along TCR affinity for both signaling nodes, with ERK1/2 phosphorylation being on average 25-fold more sensitive than pCD3ζ (Figure 4G). Finally, signal transduction and amplification from proximal pCD3ζ to distal pERK1/2 was not only stronger, but also more sustained within the optimal TCR affinity window (DMβ, TMβ) (Figure 4H; Figure S2E in Supplementary Material). This result shows that optimal TCR-pMHC interactions are able to generate significant ERK1/2 phosphorylation despite relatively low levels of pCD3ζ activation (e.g., at antigen concentration 10^−1^ µM) (Figure 4H), in agreement with the model of positive forward loops acting through ERK1/2 responses (31).

In summary, these titration experiments indicate that TCR affinity and antigen dose-associated mechanisms differentially affected the amplitude and amplification-gain of the TCR signaling pathway. Optimal TCR affinity-mediated signaling responses correlated with highest pCD3ζ and pERK1/2 activation levels as well as with greatest pCD3ζ to pERK1/2 amplification-gain for all tested antigen doses. Increased antigen doses did not allow the recovery of maximal pCD3ζ and pERK1/2 levels for low and very high TCR affinity T cells, leading to reduced signal amplitude and amplification-gain at the distal ERK1/2 signaling node. These data further support the hypothesis of an active regulatory mechanism present in the very high affinity CD8 T cells and impinging on the ERK1/2-signaling node, providing an explanation for the impaired proliferation capacity observed in these cell variants.

### Pharmacological Inhibition of SHP-1/SHP-2 Phosphatase Activity Differentially Impacts Proximal and Distal TCR Signaling Nodes, Depending on TCR Affinity

**Figure 5:**
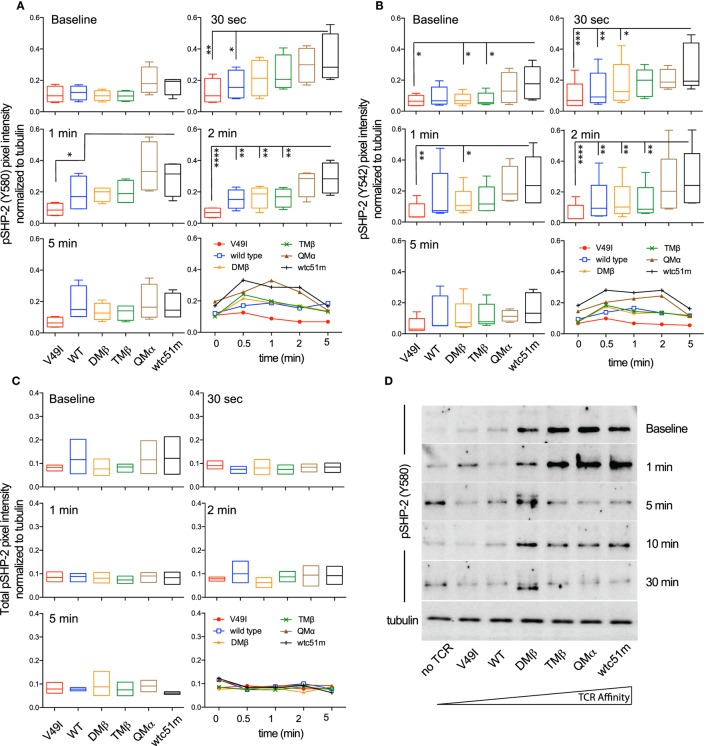
Temporal quantification of SHP-2 phosphorylation levels in T cells engineered with T cell receptor (TCR) of incremental affinities. **(A–C)** Relative intensity of **(A)** pSHP-2(Y580) (*n* = 4 independent experiments), **(B)** pSHP-2(Y542) (*n* = 5), and **(C)** total SHP-2 (*n* = 3) levels obtained by RPPA at baseline and at the indicated time-points after stimulation of the SUP-T1 cells with NY-ESO-1-specific unlabeled multimers. Data are depicted as box (25th and 75th percentile) and whisker (min to max) with the middle line representing the median. Each TCR variant is depicted by distinct color codes. Statistical analyses were performed with matched, one-way ANOVA tests followed by Dunnett’s multiple comparisons. Significance of the adjusted *p* value at α = 0.05 is given as following: *p ≤ 0.05, **p ≤ 0.01, ***p ≤ 0.001, ****p ≤ 0.0001. **(D)** Levels of pSHP-2(Y580) of TCR-transduced SUP-T1 variants at baseline and at the indicated time-points after stimulation with NY-ESO-1-specific unlabeled multimers by Western blotting. Alpha-tubulin expression levels were used as loading controls between samples. Data are representative of three independent experiments.

SHP-1 and SHP-2 phosphatases play critical roles in regulating peripheral CD8 T cell responses, notably by controlling TCR-mediated cell signaling and function (23, 34, 35). We previously reported gradual enhanced levels of SHP-1 phosphorylation [pY536 (36)] with increasing TCR affinities in primary CD8 T cells (25). Here, using the RPPA quantitative approach, we confirmed that following antigen-specific stimulation, SHP-1 phosphorylation was increased in the very high affinity SUP-T1 cells compared to optimal and low affinity TCR variants (Figure S4 in Supplementary Material). Extending these observations, we now demonstrate a rapid (30 s) and TCR affinity-associated upregulation for the two major phospho-forms of SHP-2 (pY580 and pY524) (37) (Figures 5A,B), contrasting with the stable levels observed for total SHP-2 (Figure 5C). Western blot analyses further revealed that highest levels of SHP-2 phosphorylation were readily found at baseline in high affinity T cells (Figure 5D), in line with the results found for SHP-1 activation levels (25).

**Figure 6:**
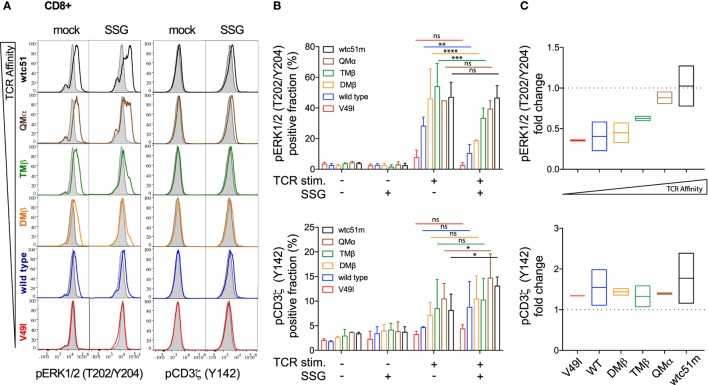
Effect of sodium stibogluconate (SSG) on proximal CD3ζ and distal ERK1/2 phosphorylation levels in T cell receptor (TCR)-engineered primary CD8 T cells. **(A)** Representative histograms of the phosphorylation levels [geometric mean fluorescence intensity (gMFI)] of ERK1/2 (left) and CD3**ζ** (Y142) (right) by phospho-flow at baseline (gray) and 5 min poststimulation with NY-ESO-1-specific unlabeled multimers (color) of TCR-transduced primary CD8 T cell variants pretreated (SSG) or not (mock) with SSG. **(B)** Quantification of the positive fraction of pERK1/2 (upper panel) and pCD3**ζ** (Y142) (lower panel) in the indicated TCR-transduced CD8 T cell variants, pretreated (SSG, +) or not (SSG, −) with SSG, at baseline (TCR stim., −) or after 5 min of stimulation (TCR stim., +) with NY-ESO-1-specific unlabeled multimer (*n* = 2 independent experiments). Statistical analyses were performed with matched, two-way ANOVA tests followed by Tukey’s multiple comparisons. Significance of the adjusted *p* value at α = 0.05 is given by the following symbols: ns *p* > 0.05 and *p ≤ 0.05, **p ≤ 0.01, ***p ≤ 0.001, ****p ≤ 0.0001. **(C)** SSG-mediated fold change in the fraction of ERK1/2 (upper panel) and CD3**ζ** (Y142) (lower panel) phosphorylation levels for the indicated TCR-transduced CD8 T cell variants after 5 min stimulation. Data are depicted as box (min to max) with the middle line representing the mean. **(A–C)** Each TCR variant is depicted by distinct color codes.

To investigate whether SHP-1 and SHP-2 activity could proportionately impact TCR-mediated signaling, SUP-T1 and primary CD8 T cells were pretreated with the SHP-1/SHP-2 inhibitor SSG, at a concentration expected to inhibit both phosphatase activities (30). We assessed the activation levels of CD3ζ and ERK1/2 before and after antigen-specific TCR stimulation. Under steady-state conditions, both pCD3ζ and pERK1/2 baseline levels remained stable and mostly unaffected by the SSG-mediated phosphatase blockade. Upon antigen-specific stimulation, pharmacological inhibition of SHP-1/SHP-2 activity profoundly affected ERK1/2 phosphorylation and to a less extent pCD3ζ (Y142), in a TCR affinity-dependent manner (Figures 6A,B; Figure S5 in Supplementary Material). Specifically, the potential of ERK1/2 phosphorylation (i.e., the fraction of pERK-responding cells and its fold change in the presence versus absence of SSG) was reduced in low (V49I, wild-type) and optimal (DMβ, TMβ) affinity TCR variants (Figures 6B,C; Figures S5B,C in Supplementary Material). In contrast, the very high (QMβ, wtc51m) affinity variants showed a better resistance to the inhibitory effect of the drug, because treated SUP-T1 and CD8 T cells were able to maintain stable pERK1/2 levels. Thus, despite an overall reduced ERK1/2 phosphorylation, relative changes in ERK1/2 activation following SSG treatment now increased with the TCR affinity gradient after antigenic stimulation, with greatest levels being found in the very high TCR variants. Interestingly, SSG treatment had an opposite effect on pCD3ζ (Y142) levels, with a modest increase in all variants (Figures 6B,C; Figure S5B,C in Supplementary Material). Yet, this trend was more pronounced in the very high affinity T cells. Together, our data suggest that SHP-1 and/or SHP-2 activity may have a distinct impact on the proximal and distal TCR signaling nodes, and this was dependent on TCR-pMHC affinity.

### SHP-1 Phosphatase Activity Negatively Regulates Proximal and Distal TCR Signaling in a TCR Affinity-Dependent Manner

**Figure 7:**
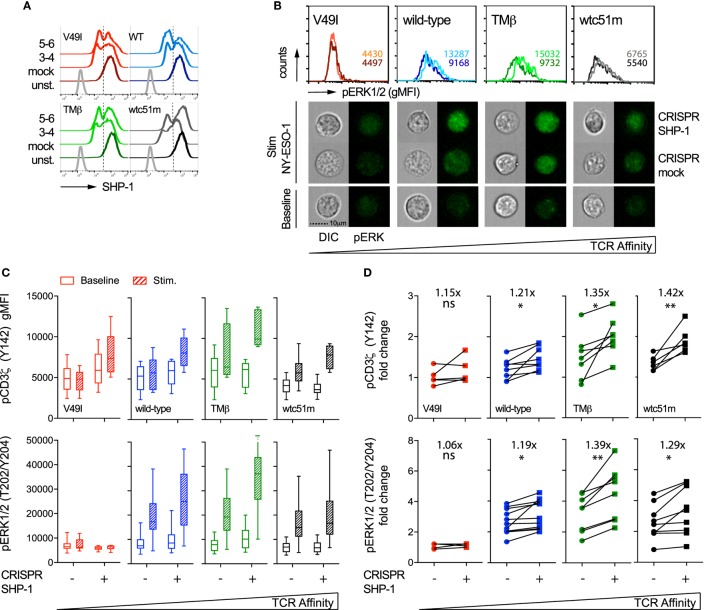
Effect of partial SHP-1 knock-out by CRISPR/Cas9 on proximal pCD3ζ and distal pERK1/2 signal intensity. **(A)** Representative histograms of the expression levels [geometric mean fluorescence intensity (gMFI)] of total SHP-1 in T cell receptor (TCR)-transduced SUP-T1 variants following CRISPR/Cas9-mediated GFP (mock) or SHP-1 [single guide RNA (sgRNA) no 3–4 and no 5–6] targeting. Unstained (unst.) controls are shown alongside. **(B)** Representative quantitative (histograms in gMFI) and qualitative (cells) data acquired with the ImageStream flow cytometer depicting the expression levels of pERK1/2 in TCR-transduced SUP-T1 variants following CRISPR/Cas9-mediated GFP mock or SHP-1 targeting. Values within the histograms indicate the average expression levels (in gMFI) of pERK1/2 following CRISPR/Cas 9-mediated GFP mock (dark) and SHP-1 (dark) targeting after stimulation with NY-ESO-1-specific unlabeled multimers. **(C)** Quantification of the total phosphorylation levels (gMFI) of CD3**ζ** (Y142) (upper panels, *n* > 4 independent experiments) and ERK1/2 (lower panels, *n* > 4 independent experiments) by phospho-flow for the indicated TCR-transduced SUP-T1 variants following CRISPR/Cas9-mediated GFP (mock; −) or SHP-1 (no 5–6; +) targeting, at baseline or after 5 min of stimulation with NY-ESO-1-specific unlabeled multimers. Data are depicted as box (25th and 75th percentile) and whisker (min to max) with the middle line representing the median. **(D)** Comparison of the relative differences (fold change phosphorylation levels between baseline and 5 min of stimulation) of pCD3**ζ** (Y142) (upper panels) and pERK1/2 (lower panels) for the indicated TCR-transduced SUP-T1 variants following CRISPR/Cas9-mediated mock (−) and SHP-1 (+) targeting. Fold change (average) in phosphorylation intensity due to SHP-1 targeting is indicated for all conditions. Statistical analyses were performed with two-tailed, paired *t* tests. Significance of the *p* value at α = 0.05 is given by the following symbols: ns *p* > 0.05 and *p ≤ 0.05, and **p ≤ 0.01. **(A–D)** Each TCR variant is depicted by a distinct color code.

**Figure 8:**
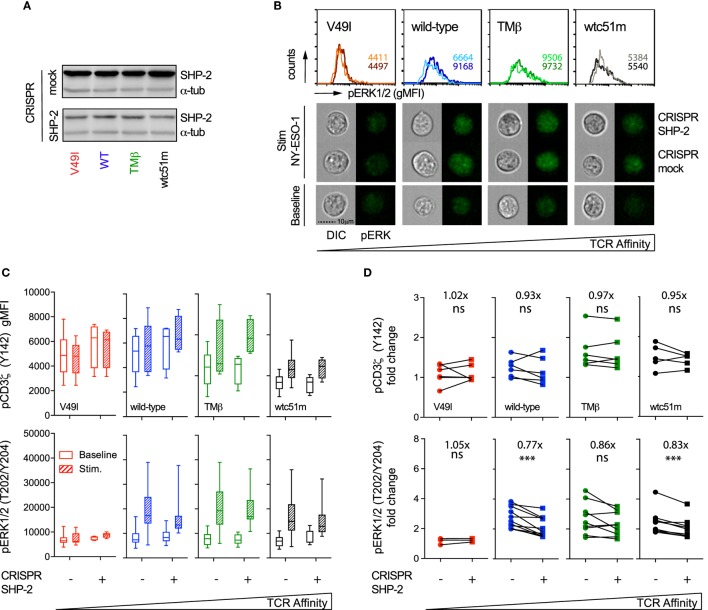
Effect of partial SHP-2 knock-out by CRISPR/Cas9 on proximal pCD3ζ and distal pERK1/2 signal intensity. **(A)** Representative Western blots of the expression level of total SHP-2 in T cell receptor (TCR)-transduced SUP-T1 variants following CRISPR/Cas9-mediated GFP (mock) or SHP-2 (sgRNA no 9–10) targeting. Alpha-tubulin expression control is shown alongside. **(B)** Representative quantitative (histograms in gMFI) and qualitative (cells) data acquired with the ImageStream flow cytometer depicting the expression levels of pERK1/2 in TCR-transduced SUP-T1 variants following CRISPR/Cas9-mediated GFP mock or SHP-2 targeting. Values within the histograms indicate the average expression levels (in gMFI) of pERK1/2 following CRISPR/Cas 9-mediated GFP mock (dark) and SHP-2 (dark) targeting after stimulation with NY-ESO-1-specific unlabeled multimers. **(C)** Quantification of the total phosphorylation levels (gMFI) of CD3**ζ** (Y142) (upper panels, *n* > 4 independent experiments) and ERK1/2 (lower panels, *n* > 4 independent experiments) by phospho-flow for the indicated TCR-transduced SUP-T1 variants following CRISPR/Cas9-mediated GFP (mock; −) or SHP-2 (+) targeting, at baseline or after 5 min of stimulation with NY-ESO-1-specific unlabeled multimers. Data are depicted as box (25th and 75th percentile) and whisker (min to max) with the middle line representing the median. **(D)** Comparison of the relative differences (fold change phosphorylation levels between baseline and 5 min of stimulation) of pCD3**ζ** (Y142) (upper panels) and pERK1/2 (lower panels) for the indicated TCR-transduced SUP-T1 variants following CRISPR/Cas9-mediated mock (−) and SHP-2 (+) targeting. Fold change (average) in phosphorylation intensity due to SHP-2 targeting is indicated for all conditions. Statistical analyses were performed with two-tailed, paired *t* tests. Significance of the *p* value at α = 0.05 is given by the following symbols: ns *p* > 0.05 and ***p ≤ 0.001. **(A–D)** Each TCR variant is depicted by a distinct color code.

In order to specifically dissect the respective contributions of SHP-1 and SHP-2 phosphatase activity to the TCR signaling pathway, we next targeted SHP-1 and SHP-2 independently with sequence-specific CRISPR/Cas9 lentiviral constructs. We obtained a knock-out phenotype in a fraction of SUP-T1 cells, thus resulting in populations with partial deficiency of SHP-1 (45–60%) and SHP-2 (60–80%) expression (Figures 7A and 8A). Since SHP-1 knock-out induced a high proportion of cell death, in agreement with a recent report (34), this limited our analyses to a relatively short-time window (~7–10 days) after selection of the CRISPR/Cas9-targeted cells. We first evaluated the impact of SHP-1 knock-out on ERK1/2 phosphorylation by directly visualizing individual SUP-T1 cells using an imaging flow cytometer that enables the qualitative selection of living cells combined to the quantitative analysis of signaling intensity (38). Upon antigen-specific stimulation, we found that ERK1/2 phosphorylation fluorescence was clearly augmented for WT, TMβ, and wtc51m-transduced SUP-T cells, but not for V49I cells, when compared to mock-targeted controls (Figure 7B).

Quantifications by phospho-flow confirmed and extended these results (Figure 7C). Indeed, partial SHP-1 knock-out resulted in the upregulation of both pCD3ζ and pERK1/2 signaling molecules in all variants, with the exception of the low affinity V49I T cells. This led to a significant increase of the amplitude of CD3ζ phosphorylation (i.e., fold change between baseline and activated state) along the TCR affinity gradient with highest fold increase found in optimal and very high affinity T cells (Figure 7D). In the latter cells, this effect was in part due to lower intensity of pCD3ζ at baseline (Figure 7C). The impact of partial SHP-1 knock-out on the amplitude of pERK1/2 was also influenced by TCR affinity, as both TMβ and wtc51m T cell variants displayed again the strongest fold change (Figure 7D).

### SHP-2 Phosphatase Activity Sustains ERK1/2 Phosphorylation but Does Not Impact Proximal TCR/CD3ζ Signal Initiation

Similar analyses were performed in SUP-T1 cell variants following CRISPR/Cas9-mediated targeting of SHP-2 phosphatase. Partial SHP-2 knock-out did not induce cell death, but SUP-T1 cells were generally larger in size than their mock-transduced counterparts (data not shown). In sharp contrast to SHP-1, partial depletion of SHP-2 led to reduced ERK1/2 phosphorylation levels in most TCR variants upon TCR stimulation (Figures 8B,C). This resulted in a clear decline in pERK1/2 amplitude (i.e., fold change) in all TCR variants, except in V49I cells (Figure 8D). However, no significant effect of SHP-2 deficiency could be detected on the intensity and amplitude of proximal CD3ζ phosphorylation compared to the respective mock controls (Figures 8C,D).

Altogether, CRISPR/Cas9 knock-out experiments revealed that SHP-1 activity mainly controls the amplitude of signaling by restricting pCD3ζ and pERK1/2 activation in a TCR affinity-dependent manner after TCR-specific activation. On the other hand, our results indicate a more global positive role of SHP-2 phosphatase on the TCR signaling pathway by sustaining downstream ERK1/2 MAPK activity, without directly impacting on TCR/CD3ζ phosphorylation. These data are in agreement with the differential impact observed on proximal versus distal TCR signaling molecules upon pharmacological SHP-1/2 phosphatase inhibition (Figure 6; Figure S5 in Supplementary Material). Thus, SHP-1 and SHP-2 phosphatase activities present distinct roles on the proximal (CD3ζ) and distal (ERK1/2) nodes of the TCR signaling pathway, and this was partially dependent on the TCR-ligand affinity.

## Discussion

T cell receptor-mediated signaling can be tuned by various regulatory loops to transmit the surface-based inputs of TCR-pMHC interactions into integrated T cell responses. During T cell development, both TCR-ligand affinity and antigen dose have been shown to induce minute differences in the physicochemical properties of the binding strength leading in turn into large changes in T cell responsiveness and fate (39). The importance of TCR-pMHC binding strengths on the magnitude of T cell responses was also demonstrated in numerous studies based on artificial models (e.g., using affinity-improved TCR variants or altered peptide ligands), highlighting that maximal T cell activation and effectiveness occur at intermediate/optimal TCR-pMHC binding affinities or half-lives (3, 4, 6, 8, 25, 33, 40–44). Thus, it was proposed that engineered TCRs against self/tumor antigens might not need to be enhanced beyond the physiological TCR affinity range (*K*_D_ < 1 µM) to achieve best T cell function (45, 46). Yet, there still is a need to improve our understanding of the underlying TCR signaling characteristics (i.e., amplitude, amplification-gain, duration) involved in generating an affinity-optimized CD8 T cell response and to study how these parameters are modulated in very high affinity T cells.

Here, using the well-established panel of HLA-A*0201/NY-ESO-1_157–165_-specific TCRs of incremental affinities (3, 11), we found that SUP-T1 and primary CD8 T cells expressing TCR affinity variants in the upper natural range (*K*_D_ ≈ 1 μM) displayed the highest and most sustained CD3ζ and ERK activation levels, which was associated with maximum CD3ζ to ERK amplification-gain, irrespectively of the antigen dose (Figures 1, 2 and 4; Figure S2E in Supplementary Material). Conversely, both lower (*K*_D_ ≥ 21.4 µM) and higher affinity (*K*_D_ < 0.1 µM) TCR-transduced T cells were unable to generate strong pERK1/2 activation and amplitude, leading to reduced functional and proliferative capacities, even at high antigen dose. Temporal analysis further confirmed that the low affinity V49I SUP-T1 cells were at all time-points ineffective at generating and maintaining pCD3ζ signaling, which is likely due to the weak intrinsic binding capacity of this TCR to the cognate pMHC (3, 11). In contrast, very high affinity T cells had rapid and intense, but only transitory proximal signaling capacity (i.e., fully activated ITAM/pCD3ζ 23 kDa), in line with a report showing accelerated responses in affinity-matured T cells specific for the HLA-A0201/Tax peptide (44). Our data indicate that robust and sustained proximal TCR signaling represents a key feature for efficient MAPK/ERK amplification and subsequent maximized T cell responsiveness. This is primarily determined at the intermediate/optimized TCR affinity range. Such observations are reminiscent of the modulations observed in TCR affinity-mediated ERK signaling during thymic positive and negative selection. Positively selected thymocytes that interact with low and intermediate affinity self-pMHCs display relatively mild but sustained ERK1/2 activation (47), while thymocytes that interact too strongly with self-pMHCs have intense but only transient pERK1/2, inducing negative selection and apoptosis (48).

Attenuation of key TCR-mediated signaling molecules in relation to increased TCR-ligand affinity has been previously documented in *in vivo* mouse studies (6, 8). Sharp declines in Akt and STAT3 phosphorylation levels were related to limited IFNγ production in CD4 T cells immunized with a high affinity moth-cytochrome ligand (8). More recently, Zhong et al. showed that TCR signaling (e.g., ERK activation and calcium mobilization) and T cell functional responses against the self/tumor antigen gp-100 plateaued at a defined TCR affinity threshold (6). Interestingly, they also reported that T cell antitumor activity and autoimmunity were closely coupled, whereby increasing TCR affinity correlated with improved tumor control, but was also linked with severe ocular autoimmunity. Thus, similarly to the events observed during thymic selection processes, TCR affinity-associated regulatory mechanisms may impinge on various levels of the TCR signaling cascade, notably at the MAPK amplification node, and could possibly restrict effective T cell responses.

We further found that TCR/CD3 complex triggering by the strong agonist OKT3/CD3ε antibody was unable to restore full ERK1/2 activation levels in both SUP-T1 and CD8 T cells engineered with very high TCR affinities (Figure 1). Yet, these T cells were not ERK-defective, since TCR-independent stimulation by PMA/ionomycin induced ERK1/2 activation equally well throughout the entire panel of affinity-increased T cells. Moreover, under steady-state settings, levels of TCR/CD3 complex, CD8β and CD28 expression, as well as of phosphorylated ITAM/CD3ζ (Y142) were also substantially decreased in the very high affinity T cells [Figure 3 (25)], while the baseline levels of phosphorylated SHP-1 and SHP-2 phosphatases were highest [Figure 5 (25)]. These observations are in line with the reduced levels of coactivatory molecules HVEM and CD70, but increased expression of the inhibitory receptor PD1 detected previously in resting primary CD8 T cells with very high affinity TCRs (25). Finally, no changes in basal levels were found for total CD3ε, total LCK, pLCK(Y394/Y416), pLCK(Y505), total ZAP-70 and pZAP-70(Y319), nor for total ERK and pERK1/2 [Figure 3; Figures S1 and S2 in Supplementary Material (25)]. Together, our data indicate that the very high affinity SUP-T1 and CD8 T cells share features in common with a TCR-mediated hyporesponsive state and that TCR affinity-dependent regulatory mechanism(s) may readily be present in resting conditions. Furthermore, the TCR/CD3 complex itself seems to be under strict affinity-dependent regulation and may directly or indirectly dictate differential activation thresholds upon TCR triggering and T cell activation. Thus, inducing a TCR hyporesponsive state may be part of a physiological protection mechanism preventing harmful effects that could potentially occur in response to high affinity/avidity TCR recognition of self-antigens.

Several tolerogenic mechanisms such as anergy- or exhausted-related states may have an impact on T cell activation and responsiveness through TCR recognition of peptide/MHC. Recent findings support the notion that increasing the TCR signaling strength favors the induction of a hyporesponsive state (49, 50). For instance, it was shown in an autoimmune mouse model that T cells expressing a high TCR avidity (either through increase of TCR-pMHC affinity or antigen dose) became anergic, a cell fate decision that was imprinted by PD-1 upregulation and pERK1/2 inhibition (49). Hsu et al. further reported that peripheral CD4 T cells with a ZAP-70 gain-of-function mutation inducing enhanced basal TCR signaling displayed a T cell anergy-like phenotype with increased expression of PD-1 (50). At present, it remains unclear what causes the TCR-mediated hyporesponsive state observed in engineered CD8 T cells of very high but not intermediate TCR affinity, readily detectable under resting culture conditions. Through structure-based rational predictions, the TCR affinity-optimization process used here were achieved by the combination of point mutations within the CDR2α and/or CDR2β and/or CDR3β loops (3, 12). Specifically, TMβ and QMα TCR variants contain 2 and 3 mutations within the CDR2α/β loops, respectively. Moreover, we also included the very high affinity TCR variant wtc51m, previously identified by phage-display screening, and containing up to four mutations in the CDR2β loop (51). Importantly, our TCR affinity-improved panel remained ligand-specific and retained similar TCR-pMHC binding recognition patterns compared to the wild-type TCR (3). Therefore, one likely hypothesis is that the gain in affinity (*K*_D_ < 1 µM) within our panel of engineered T cells is mainly related to amino acid mutations within the CDR2α/β loops, mostly interacting with the HLA-A2 backbone (3). In the very high affinity T cells (*K*_D_ < 1 µM), this may trigger chronic TCR-HLA-A2 binding interactions, even in the absence of cognate peptide antigen, resulting in a hyporesponsive cellular and functional state. Preliminary results support this hypothesis, since reduced CD28 and TCR β-chain (BV13.1) expression in very high affinity TCR variants was predominantly found in HLA-A2^pos^ TCR-engineered CD8 T cells when compared to the corresponding HLA-A2^neg^ cells (Figure 3F, Duong M.-N. et al., unpublished data). This was associated with the upregulation of immune modulators such as PD-1 in HLA-A2^pos^ but not HLA-A2^neg^ engineered CD8 T cells (Duong M.-N. et al., unpublished data). Future analysis involving the careful evaluation of the molecular combined to functional regulation of this TCR affinity-dependent hyporesponsive state is necessary to understand the precise contribution of the TCR-HLA-A2-mediated interactions in our TCR affinity-increased model. Our observations are further consistent with a report (52) describing the importance of TCR-peptide/self-MHC interactions in controlling basal T cell activation levels and the ensuing T cell responsiveness to foreign antigen. Namely, in the absence of self-MHC recognition on dendritic cells, T cells became impaired in TCR/CD3ζ-mediated signaling, and consequently hyporesponsive to antigen, leading to reduced T cell proliferative responses (52). Together, we propose that adjusted TCR-peptide/self-MHC interactions might be essential in setting an optimal basal T cell activation threshold for subsequent agonist-dependent TCR signaling initiation and T cell function.

Our work offers clear evidence that SHP-1 and SHP-2 phosphatase activities have an opposite biological impact on the ERK signaling node (Figures 6–8). While reduced SHP-1 expression upon CRISPR/Cas9 revealed significant increased ERK1/2 phosphorylation, SHP-2 depletion led to a clear decline in ERK1/2 activation levels. Besides, the SHP-1-mediated effect was mostly observed in the higher affinity SUP-T1 cells, differing from the SHP-2-mediated one, which affected both physiological and supraphysiological affinity TCR variants. Similarly, SHP-1 but not SHP-2 activity had an impact on proximal TCR/CD3ζ signal initiation, in a TCR affinity-dependent manner. Indeed, we found an increase in CD3ζ activation upon partial SHP-1 knock-out, which is consistent with the known implication of SHP-1 in counteracting TCR signal initiation, likely through the direct dephosphorylation of CD3ζ (53). In contrast, no significant effect of SHP-2 deficiency was found on the intensity or the amplitude of CD3ζ phosphorylation. In light of these results, pharmacological inhibition by SSG at a concentration known to affect both SHP-1 and SHP-2 phosphatases (30) yielded comparable results in TCR-engineered primary CD8 T and SUP-T1 cells. Our data suggest that the TCR affinity-associated increase in ERK1/2 phosphorylation observed after SHP-1 inhibition (Figure 7) may compensate the general decrease of pERK1/2 detected after SHP-2 depletion (Figure 8). Following SHP-1/SHP-2 inhibition by SSG, this would result in relative pERK levels being less reduced in very high affinity T cells compared to optimal or wild-type ones (Figure 6; Figure S5 in Supplementary Material), in which the impact of SHP-1 inhibition is less prevalent. Moreover, the small yet positive increase in pCD3ζ levels along the TCR affinity gradient upon SSG treatment further argues in favor of a predominant role of SHP-1 phosphatase at the proximal TCR signaling node. Collectively, our data indicate that SHP-1 activity negatively modulates proximal (CD3ζ) and distal (ERK1/2) TCR signaling in a TCR affinity-dependent manner, while SHP-2 activity plays a more global and positive impact in promoting downstream ERK1/2 MAPK activity.

SHP-1 is generally viewed as a negative regulator of the TCR-mediated signaling cascade, whereas SHP-2 appears to play a more complex role, likely resulting from its several potential targets as well as their spatial level (i.e., proximal versus distal) within the TCR and coactivatory signaling pathways. For instance, the transient association between PD-1 receptor and SHP-2 into so-called inhibitory microclusters mediates the dephosphorylation of proximal TCR signaling molecules, in a TCR stimulation strength-dependent manner (23). A recent study further revealed that the costimulatory molecule CD28 is a preferred target over the TCR for dephosphorylation by PD-1-recruited SHP-2 phosphatase (24). This is in line with the absence of SHP-2-associated impact on CD3ζ phosphorylation in the present study, mainly because we specifically triggered the TCR signaling cascade using multimers. Our results are also in agreement with the reported positive role of SHP-2 in promoting ERK1/2 activation (21, 54, 55), although this was not directly related to the TCR affinity-dependent increase in pSHP-2 (Y542 and Y580) levels observed after TCR-specific triggering (Figure 5). Indeed, highest levels of pSHP-2 were seen in the very high affinity T cells and occurred early after stimulation (30 sec, Figure 5), but this phosphorylation pattern was not mirrored by pERK1/2 upregulation, taking place later and mainly in the optimal affinity TCR variants (≥2 min; Figure 1; Figure S1 in Supplementary Material). Several explanations could account for this observation. First, these data are in accordance to the rapid but transient proximal signals (pCD3ζ and pLCK/Src) found in T cells with very high affinity TCRs, yet resulting in poor MAPK activation and low proliferative capacity (Figure 2; Figure S2 in Supplementary Material). This was also associated with lowest pCD3ζ to pERK amplification gain in comparison to optimal TCR variants (Figure 4). Together, our findings further support the notion that TCR affinity-dependent regulatory mechanisms may upon TCR triggering, rapidly counteract downstream propagation and amplification signals, thus calibrating optimal signaling and subsequent function within the physiological TCR affinity range. An alternative explanation may come from the dual (i.e., positive versus negative) role of SHP-2 played at distinct nodes of the TCR signaling pathway (22), which may hide a potential TCR affinity-dependent impact, resulting in a more global SHP-2 activity-mediated effect on pERK1/2 (Figure 8). In contrast, SHP-1 phosphatase was found to exert a clear negative effect on the proximal CD3ζ signal activation in a TCR affinity-dependent manner, which may further translate to the distal MAPK node (Figure 7). Finally, it was recently shown that SHP-1’s phosphorylation status does not necessarily correspond to its phosphatase activity (56). Given the structural homology existing between SHP-1 and SHP-2 phosphatase (18), this may also occur for SHP-2, but remains to be determined.

Two models have been proposed to describe the molecular mechanisms involving SHP-1 phosphatase in regulating T cell output signals in relation to TCR-ligand affinity. In the first one, SHP-1 acts as a negative feedback loop in response to antagonist or weak TCR stimulation through its direct interaction with LCK, leading to subsequent inhibition of TCR signaling (35). In turn, stronger TCR activation induces an ERK-dependent phosphorylation of LCK at Ser59, blocking SHP-1 binding to LCK and enabling positive TCR signal amplification and maintenance. Interestingly, this model nicely fits with the pattern of pERK1/2 detected in our panel of CD8 T cells, whereby optimal affinity cells exhibited highest MAPK activity in contrast to low affinity T cells. Moreover, the LCK-ERK-SHP-1-mediated mechanism may also be involved in the supraphysiological affinity T cells, as preliminary data suggest that LCK-Ser59 phosphorylation pattern mirrors the pERK1/2 bell-shape one along the TCR affinity gradient (unpublished data). In fact, maximal LCK-Ser59 phosphorylation levels were found in the optimal affinity T cells, compared to low and very high affinity cells. However, in-depth temporal evaluation of ERK-mediated LCK/Ser59 phosphorylation within our affinity-increased CD8 T cell panel, as well as its functional relevance should deserve further investigations, since knock-in-targeted replacement of LCK-Ser59 by alanine did not affect TCR signaling and ligand discrimination in an *in vivo* model (34). The second model proposes that SHP-1 as well as SHP-2 constitutively interact with THEMIS, a key TCR signal regulator for ligand discrimination, which upon TCR triggering recruits both phosphatases to the LAT/GRB2 signalosome complex (34, 57). THEMIS was initially proposed to act as a negative regulator of TCR signaling *via* SHP-1 by regulating neighboring TCR signaling targets (57). However, recent studies have refined this view by showing that THEMIS may in fact have a positive effect on TCR signaling during thymocyte development, notably by suppressing SHP-1 tyrosine-phosphatase activity (56, 58). Together, these observations demonstrate a critical role of the THEMIS-SHP complex during thymocyte development as a fine-tuner of TCR signaling threshold, but whether similar regulatory mechanisms also apply within our model of affinity-improved T cells remains to be determined.

Our findings have implications in the design of affinity-improved TCRs for immunotherapy, since it is becoming clear that regulatory signaling mechanisms are associated with increasing TCR-ligand affinity in TCR-redirected CD8 T cells. Moreover, identifying the key regulatory molecules triggering TCR hyporesponsiveness in very high affinity CD8 T cells is also relevant in our understanding of T cell-mediated immune tolerance processes, as they might potentially offer new molecular targets that could be modulated during CD8 T cell responses.

## Ethics Statement

Human peripheral blood cells were obtained from healthy donors of the Blood Transfusion Center of the University of Lausanne. All donors had previously completed the Swiss National Medical questionnaire to verify that they fulfilled the criteria for blood donation and provided written informed consent for the use of blood samples in medical research after anonymization.

## Author Contributions

Study design: NR and MH. Acquisition of data: DP, EE, M-AD, MA, MQ, P-OR, and MH. Analysis and interpretation of data: DP, EE, M-AD, MA, MQ, P-OR, MD, NR, and MH. Writing, review and/or revision of the manuscript: NR and MH.

## Conflict of Interest Statement

The authors declare that the research was conducted in the absence of any commercial or financial relationships that could be construed as a potential conflict of interest.

## Supplemental Materials and Methods

### CD107a Degranulation and Intracellular Cytokine Staining Assays

Native NY-ESO-1_157-165_ peptides were incubated for 1 hour at room temperature with the disulfide-reducing agent TCEP (2 mM, Pierce Biotechnology). HLA-A*0201-positive TAP-deficient T2 cells were pulsed 1 hour at 37°C with varying doses of NY-ESO-1_157-165_, peptide, washed and incubated with TCR-transduced primary CD8 T cells at an E:T ratio of 1:2 for 4 hours in the presence of anti-CD107a/LAMP1 (FITC, clone H4A3) (BD Pharmingen) and Brefeldin A (10 µg/ml, Sigma). Cells were stained with anti-CD8α Pacific-Blue (clone B9.11) (Beckman Coulter) at 4°C for 30 min, subsequently fixed in 1% formaldehyde, 2% glucose and 5 mM NaN_3_ for 20 min at RT and permeabilized with 0.1% Saponin (Sigma Aldrich). Samples were then stained with anti-IL-2 (PerCP-Cy5.5, clone MQ1-17H12), anti-IL-13 (APC, clone JES10-5A2), anti-IFNγ (PE-Cy7, clone 4 S.B3) and anti-TNFα (Alexa Fluor700, clone Mab11) (BD Pharmingen) and anti-IL-4 (PE, clone MP4-25D2) (Biologend) for 30 min at 4°C. The samples were acquired with a Gallios (Beckman Coulter, CA, USA) flow cytometer. The data analysis was done on FlowJo (Tree star) and Prism (Graphpad, USA) platforms.

Figure S1 Analysis of the expression levels of pERK1/2 signal by the RPPA technology. **(A)** Relative intensity of ERK1/2 phosphorylation levels at baseline and at the indicated time-points after stimulation of the TCR-transduced SUP-T1 variants with NY-ESO-1-specific unlabeled multimers (*n* = 8 independent experiments). **(B)** Relative intensity of total ERK1/2 expression levels at baseline and at the indicated time-points after stimulation of the TCR-transduced SUP-T1 variants with NY-ESO-1-specific unlabeled multimers (*n* = 8 independent experiments). Statistical analyses were performed with matched, one-way ANOVA tests followed by Dunnett’s multiple comparisons. Significance of the adjusted *p* value at α = 0.05 as following: ***p* ≤ 0.01, ****p* ≤ 0.001, *****p* ≤ 0.0001. Data are depicted as box (25th and 75th percentile) and whisker (min to max) with the middle line representing the median. **(A–C)** Each TCR variant is depicted by a distinct color code.

Figure S2 Expression level of pLCK/Src(Y394/Y416), pLCK(Y505) and total LCK by the RPPA technology. **(A–C)** Relative intensity of activatory LCK/Src(Y394/Y416) phosphorylation (*n* = 4 independent experiments) **(A)**, inhibitory LCK(Y505) phosphorylation (*n* = 5 independent experiments) **(B)** and total LCK/Src expression (*n* = 5 independent experiments) **(C)** levels by RPPA at baseline and at the indicated time-points after stimulation of the TCR-transduced SUP-T1 variants with NY-ESO-1-specific unlabeled multimers. Statistical analyses were performed with matched, one-way ANOVA tests followed by Dunnett’s multiple comparisons. Data are depicted as box (25^th^ and 75^th^ percentile) and whisker (min to max) with the middle line representing the median. **(D)** Quantification of the phosphorylation levels (in gMFI) of unstimulated, baseline CD3**ζ** (Y142) and ERK1/2(T202/Y204) for the indicated TCR-transduced primary CD8 T cell variants. Data are representative of 6–12 independent experiments. **(E)** Quantification of the signal amplification gain (relative fold increase) from pCD3**ζ** (Y142) to pERK1/2 following 30 min after stimulation of the indicated TCR-transduced SUP-T1 variants with NY-ESO-1-specific unlabeled multimers. **(A–E)** Each TCR variant is depicted by the same distinct color code.

Figure S3 Antigen dose titration experiments of degranulation and cytokine production in TCR-transduced primary CD8 T lymphocytes. **(A)** Representative histograms of an intracellular staining experiment showing the intensity of degranulation (CD107a/LAMP1) and the expression levels of IFNγ, TNFα, IL-2, IL-4 and IL-13 in primary CD8 T cells transduced with an independent panel of affinity-optimized TCRs (T2A linked alpha and beta TCR panel) after 4 hours of incubation with TAP-deficient T2 cells pulsed with graded amounts of native NY-ESO-1_157-165_ peptide. (*n* = 4 independent experiments) **(B)** Quantification of the CD107a/LAMP1 degranulation intensity and the expression levels of the indicated cytokines. Data obtained from the different antigen doses (0.01, 0.1, 1, and 10 µM) are depicted as individual curves.

Figure S4 Expression level of pSHP-1(Y536) and total SHP-1 by the RPPA technology. **(A,B)** Relative intensity of SHP-1(Y536) phosphorylation (*n* = 5 independent experiments) **(A)** and total SHP-1 expression (*n* = 3 independent experiments) **(B)** levels at baseline and at the indicated time-points after stimulation of the TCR-transduced SUP-T1 variants with NY-ESO-1-specific unlabeled multimers. Statistical analyses were performed with matched, one-way ANOVA tests followed by Dunnett’s multiple comparisons. **p* ≤ 0.05, ***p* ≤ 0.01, ****p* ≤ 0.001.

Figure S5 Effect of sodium stibogluconate (SSG) on proximal pCD3ζ and distal pERK1/2 signal intensity in TCR-transduced SUP-T1 cells. **(A)** Representative histograms of the phosphorylation levels (gMFI) of ERK1/2 (left) and CD3**ζ** (Y142) (right) by phospho-flow at baseline (gray) and 5 min poststimulation with NY-ESO-1-specific unlabeled multimers (color) of TCR-transduced SUP-T1 cells pretreated (SSG) or not (mock) with sodium stibogluconate. **(B)** Quantification of the positive fraction of pERK1/2 (upper panel) and pCD3**ζ** (Y142) (lower panel) in the indicated TCR-transduced SUP-T1 cells, pretreated (SSG, +) or not (SSG, −) with sodium stibogluconate, at baseline (TCR stim., −) or after 5 min of stimulation (TCR stim., +) with NY-ESO-1-specific unlabeled multimers (*n* = 4 independent experiments). Statistical analyses were performed with matched, two-way ANOVA tests followed by Tuckey’s multiple comparisons. Significance of the adjusted *p* value at α = 0.05 is given by the following symbols: ns *p* > 0.05 and **p* ≤ 0.05, ***p* ≤ 0.01, ****p* ≤ 0.001, *****p* ≤ 0.0001. **(C)** SSG-mediated fold change in the fraction of ERK1/2 (upper panel) and CD3**ζ** (Y142) (lower panel) phosphorylation levels for the indicated TCR-transduced SUP-T1 cells after 5 min of stimulation. Data are depicted as box (min to max) with the middle line representing the mean. **(A–C)** Each TCR variant is depicted by a distinct color code.

## Abbreviations

TCR, T cell receptor; pMHC, peptide-major histocompatibility complex; A2, HLA-A*0201; LCK, lymphocyte-specific protein tyrosine kinase; ZAP-70, zeta chain of T cell receptor-associated protein kinase 70; LAT, linker for activation of T cells; SLP-76, SH2 domain-containing leukocyte protein of 76 kDa; MAPK, mitogen-activated protein kinases; PTPN6/SHP-1 and PTPN11/SHP-2, protein tyrosine phosphatase, non-receptor type 6 and type 11.
