# Supplementary figures and images for: Fine-Tuning of Optimal TCR Signaling in Tumor-Redirected CD8 T Cells by Distinct TCR Affinity-Mediated Mechanisms
Source: Front Immunol. 2017 Nov 15;8:1564. doi: 10.3389/fimmu.2017.01564 (PMC5694758; doi:10.3389/fimmu.2017.01564)

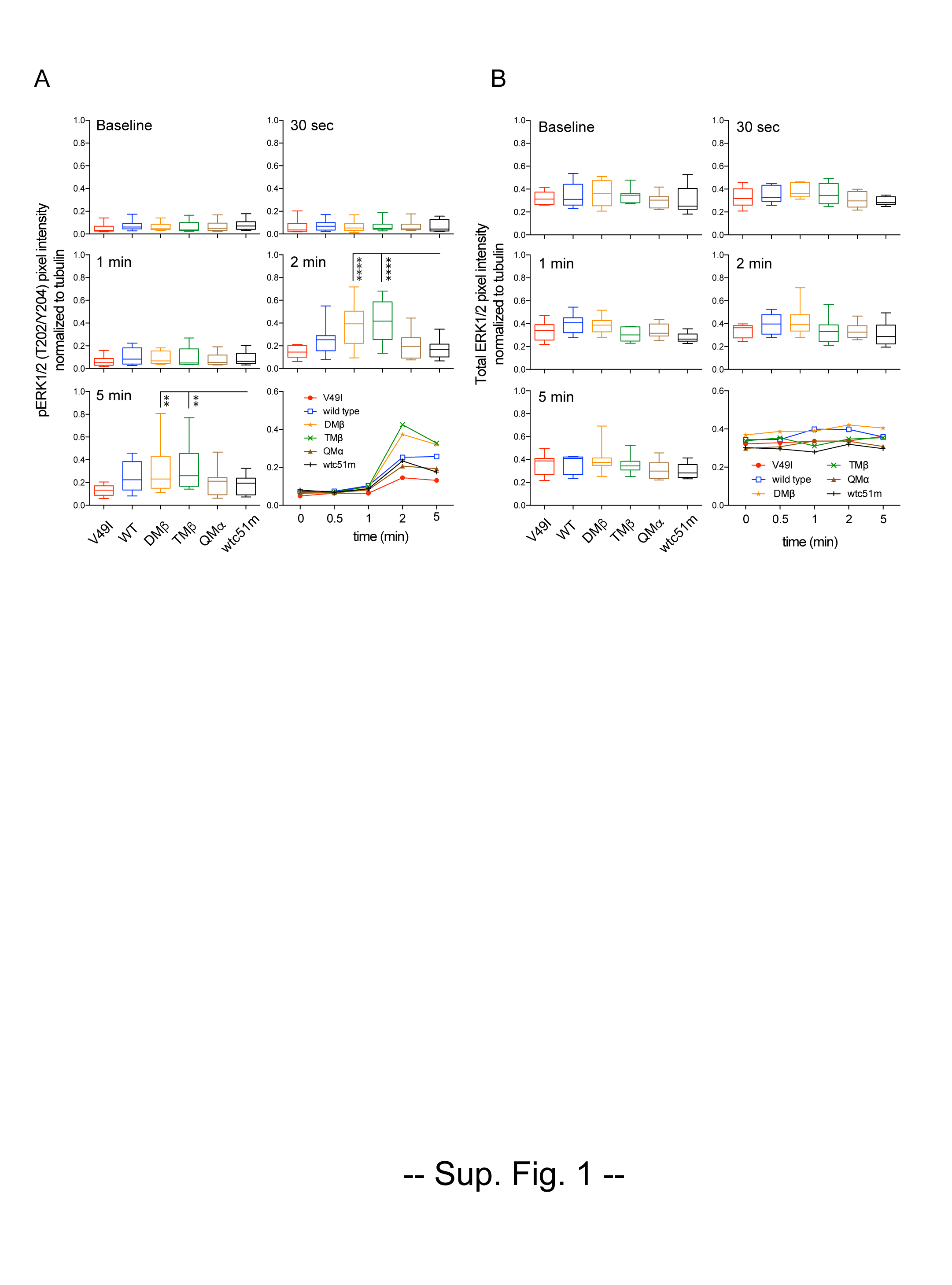

Supplement: Figure S1 — Analysis of the expression levels of pERK1/2 signal by the RPPA technology. (A) Relative intensity of ERK1/2 phosphorylation levels at baseline and at the indicated time-points after stimulation of the TCR-transduced SUP-T1 variants with NY-ESO-1-specific unlabeled multimers (n = 8 independent experiments). (B) Relative intensity of total ERK1/2 expression levels at baseline and at the indicated time-points after stimulation of the TCR-transduced SUP-T1 variants with NY-ESO-1-specific unlabeled multimers (n = 8 independent experiments). Statistical analyses were performed with matched, one-way ANOVA tests followed by Dunnett’s multiple comparisons. Significance of the adjusted p value at α = 0.05 as following: **p ≤ 0.01, ***p ≤ 0.001, ****p ≤ 0.0001. Data are depicted as box (25th and 75th percentile) and whisker (min to max) with the middle line representing the median. (A–C) Each TCR variant is depicted by a distinct color code. [file Image_1.tif]

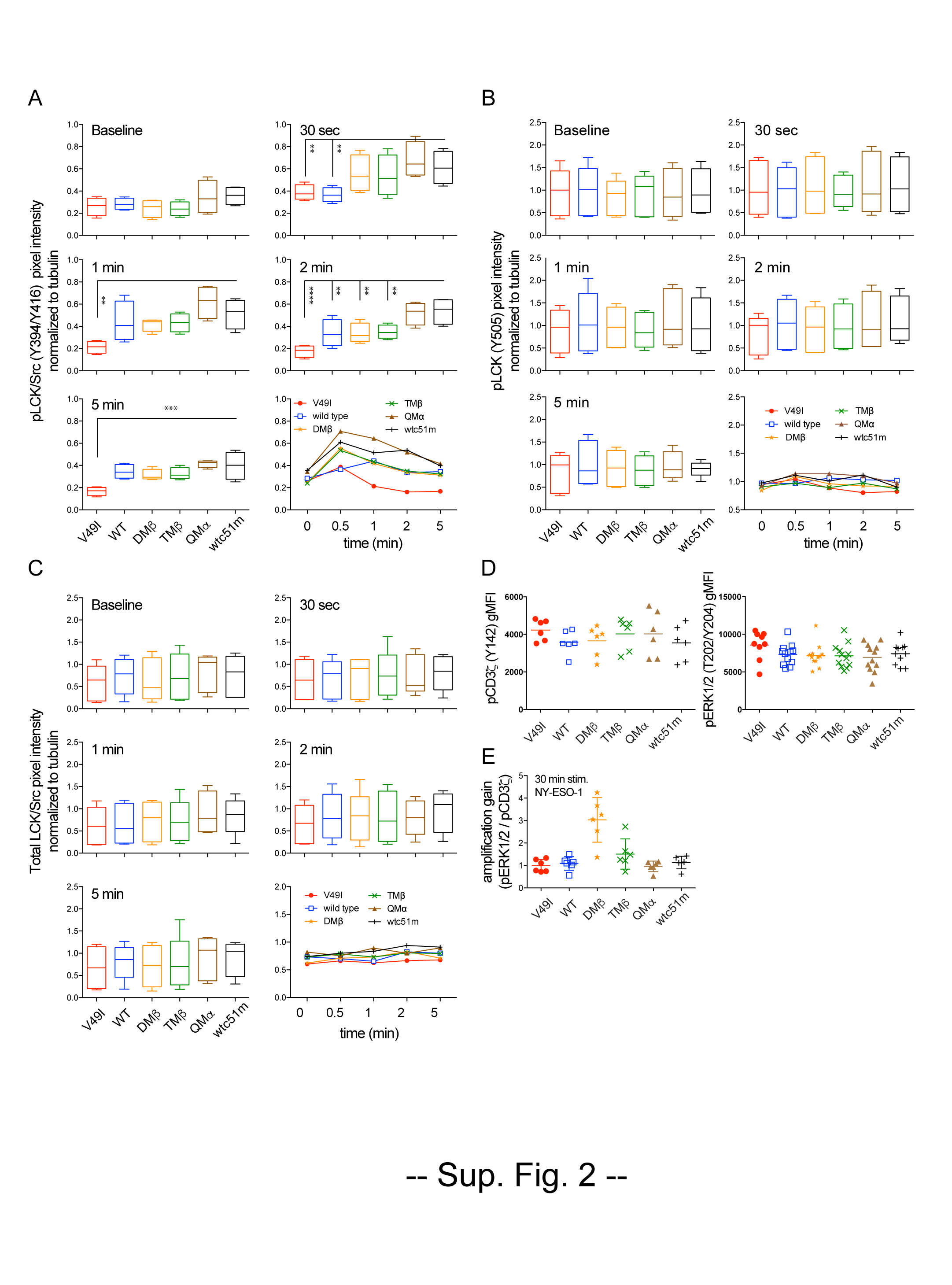

Supplement: Figure S2 — Expression level of pLCK/Src(Y394/Y416), pLCK(Y505) and total LCK by the RPPA technology. (A–C) Relative intensity of activatory LCK/Src(Y394/Y416) phosphorylation (n = 4 independent experiments) (A), inhibitory LCK(Y505) phosphorylation (n = 5 independent experiments) (B) and total LCK/Src expression (n = 5 independent experiments) (C) levels by RPPA at baseline and at the indicated time-points after stimulation of the TCR-transduced SUP-T1 variants with NY-ESO-1-specific unlabeled multimers. Statistical analyses were performed with matched, one-way ANOVA tests followed by Dunnett’s multiple comparisons. Data are depicted as box (25th and 75th percentile) and whisker (min to max) with the middle line representing the median. (D) Quantification of the phosphorylation levels (in gMFI) of unstimulated, baseline CD3ζ (Y142) and ERK1/2(T202/Y204) for the indicated TCR-transduced primary CD8 T cell variants. Data are representative of 6–12 independent experiments. (E) Quantification of the signal amplification gain (relative fold increase) from pCD3ζ (Y142) to pERK1/2 following 30 min after stimulation of the indicated TCR-transduced SUP-T1 variants with NY-ESO-1-specific unlabeled multimers. (A–E) Each TCR variant is depicted by the same distinct color code. [file Image_2.tif]

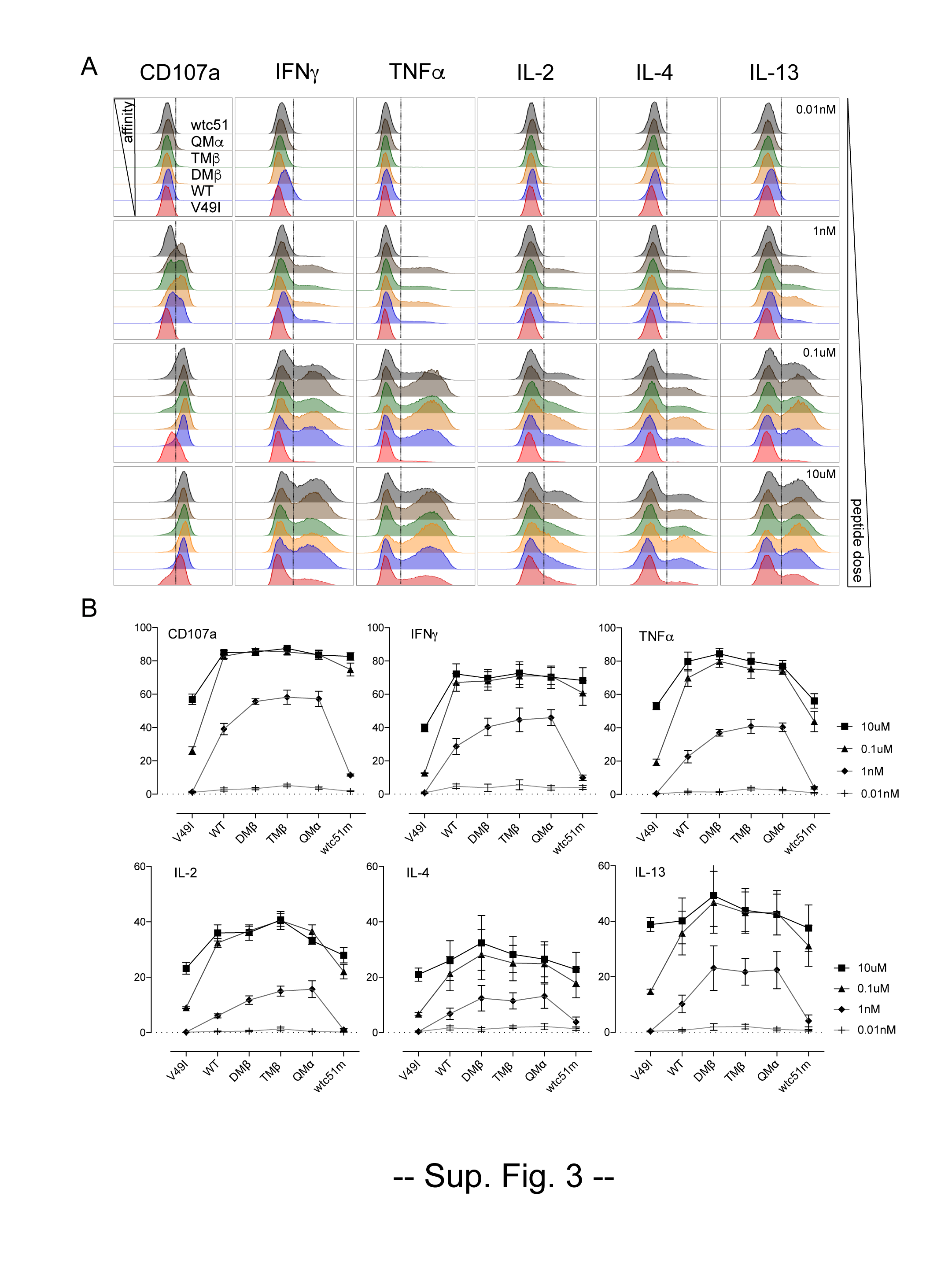

Supplement: Figure S3 — Antigen dose titration experiments of degranulation and cytokine production in TCR-transduced primary CD8 T lymphocytes. (A) Representative histograms of an intracellular staining experiment showing the intensity of degranulation (CD107a/LAMP1) and the expression levels of IFNγ, TNFα, IL-2, IL-4 and IL-13 in primary CD8 T cells transduced with an independent panel of affinity-optimized TCRs (T2A linked alpha and beta TCR panel) after 4 hours of incubation with TAP-deficient T2 cells pulsed with graded amounts of native NY-ESO-1157-165 peptide. (n = 4 independent experiments) (B) Quantification of the CD107a/LAMP1 degranulation intensity and the expression levels of the indicated cytokines. Data obtained from the different antigen doses (0.01, 0.1, 1, and 10 µM) are depicted as individual curves. [file Image_3.tif]

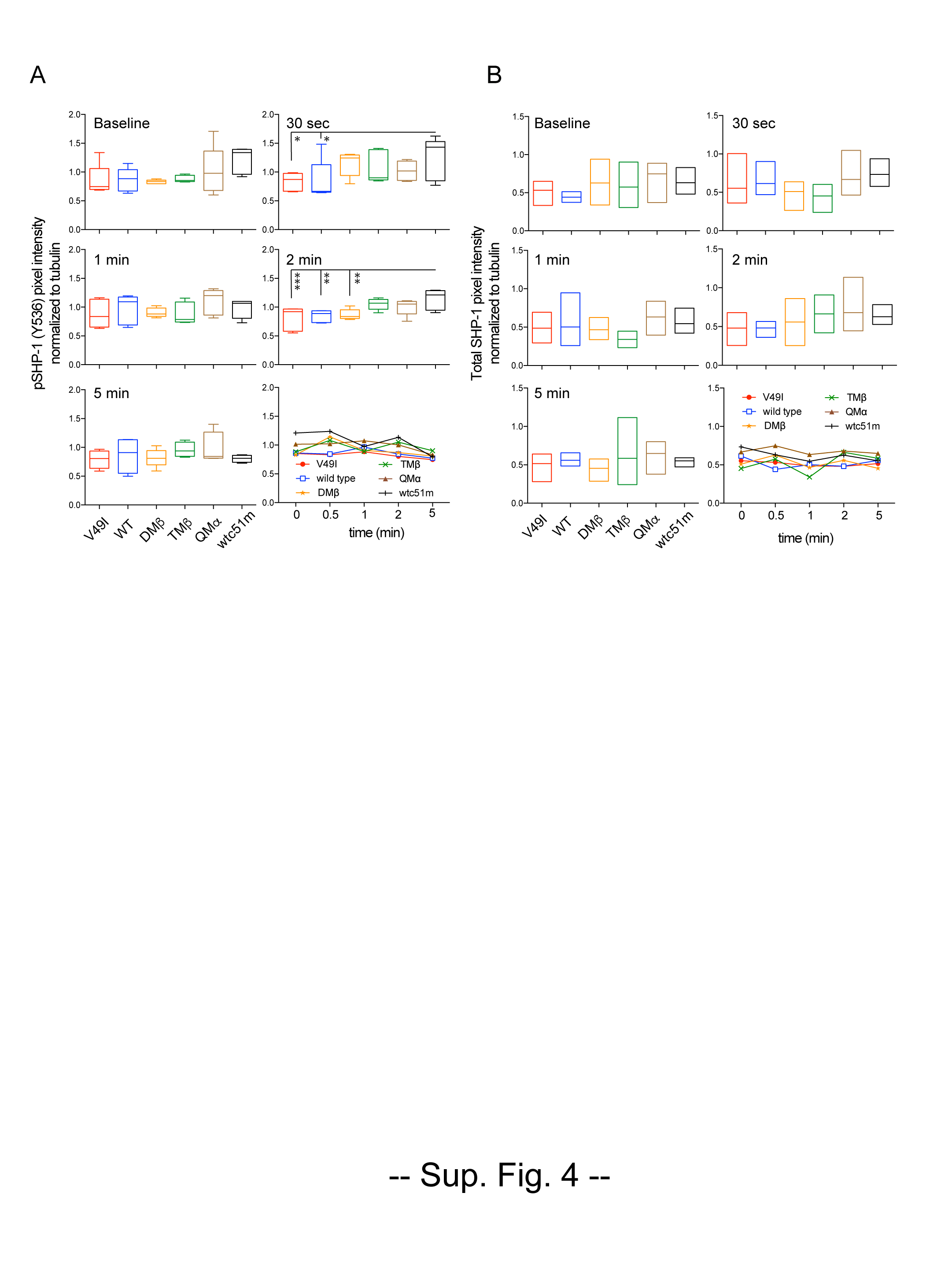

Supplement: Figure S4 — Expression level of pSHP-1(Y536) and total SHP-1 by the RPPA technology. (A,B) Relative intensity of SHP-1(Y536) phosphorylation (n = 5 independent experiments) (A) and total SHP-1 expression (n = 3 independent experiments) (B) levels at baseline and at the indicated time-points after stimulation of the TCR-transduced SUP-T1 variants with NY-ESO-1-specific unlabeled multimers. Statistical analyses were performed with matched, one-way ANOVA tests followed by Dunnett’s multiple comparisons. *p ≤ 0.05, **p ≤ 0.01, ***p ≤ 0.001. [file Image_4.tif]

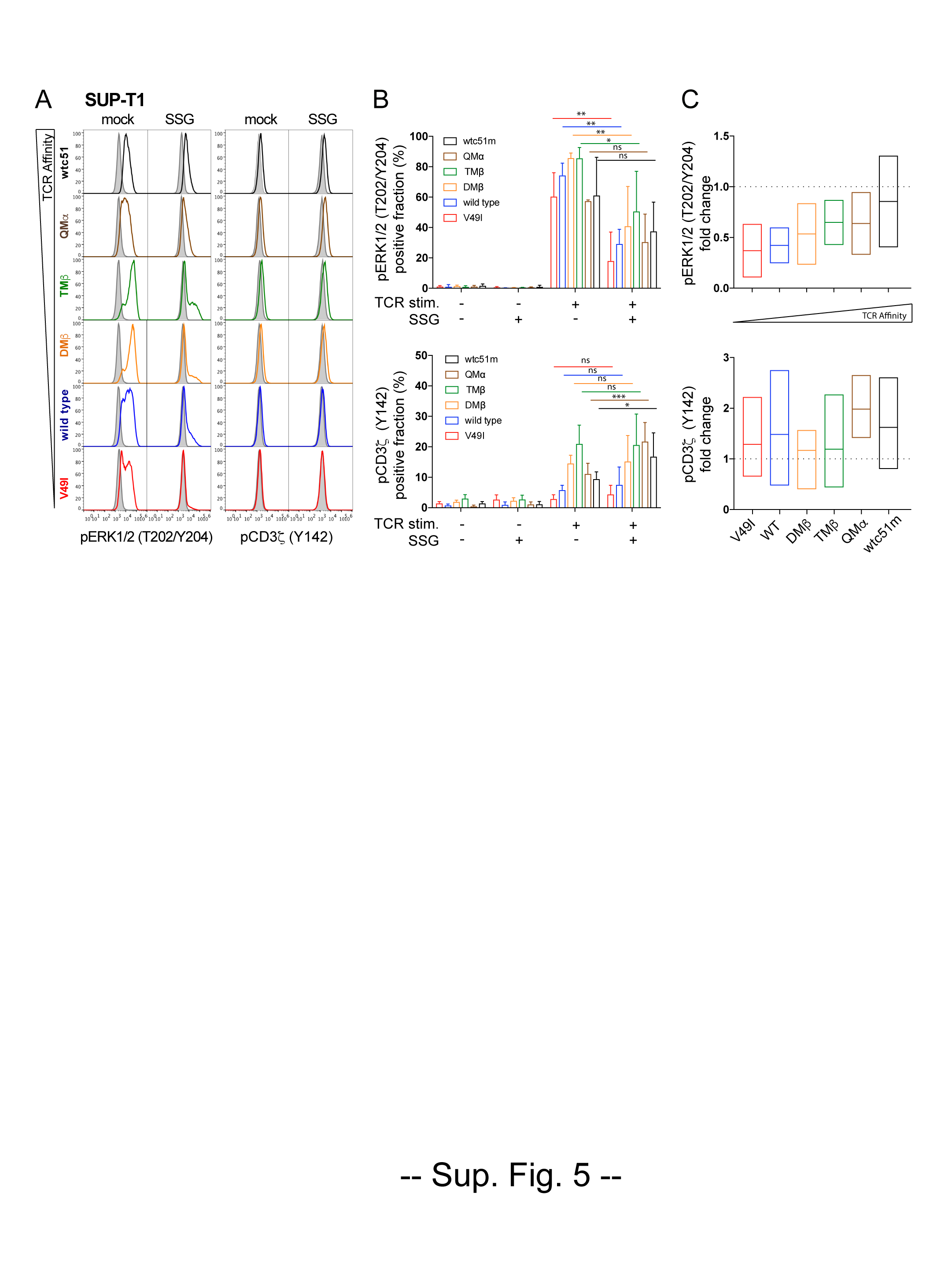

Supplement: Figure S5 — Effect of sodium stibogluconate (SSG) on proximal pCD3ζ and distal pERK1/2 signal intensity in TCR-transduced SUP-T1 cells. (A) Representative histograms of the phosphorylation levels (gMFI) of ERK1/2 (left) and CD3ζ (Y142) (right) by phospho-flow at baseline (gray) and 5 min poststimulation with NY-ESO-1-specific unlabeled multimers (color) of TCR-transduced SUP-T1 cells pretreated (SSG) or not (mock) with sodium stibogluconate. (B) Quantification of the positive fraction of pERK1/2 (upper panel) and pCD3ζ (Y142) (lower panel) in the indicated TCR-transduced SUP-T1 cells, pretreated (SSG, +) or not (SSG, −) with sodium stibogluconate, at baseline (TCR stim., −) or after 5 min of stimulation (TCR stim., +) with NY-ESO-1-specific unlabeled multimers (n = 4 independent experiments). Statistical analyses were performed with matched, two-way ANOVA tests followed by Tuckey’s multiple comparisons. Significance of the adjusted p value at α = 0.05 is given by the following symbols: ns p > 0.05 and *p ≤ 0.05, **p ≤ 0.01, ***p ≤ 0.001, ****p ≤ 0.0001. (C) SSG-mediated fold change in the fraction of ERK1/2 (upper panel) and CD3ζ (Y142) (lower panel) phosphorylation levels for the indicated TCR-transduced SUP-T1 cells after 5 min of stimulation. Data are depicted as box (min to max) with the middle line representing the mean. (A–C) Each TCR variant is depicted by a distinct color code. [file Image_5.tif]
